# Development and Piloting of Implementation Strategies to Support Delivery of a Clinical Intervention for Postpartum Hemorrhage in Four sub-Saharan Africa Countries

**DOI:** 10.9745/GHSP-D-23-00387

**Published:** 2024-10-29

**Authors:** Gillian Forbes, Shahinoor Akter, Suellen Miller, Hadiza Galadanci, Zahida Qureshi, Fadhlun Alwy Al-beity, G. Justus Hofmeyr, Neil Moran, Sue Fawcus, Mandisa Singata-Madliki, Aminu Ado Wakili, Taiwo Gboluwaga Amole, Baba Maiyaki Musa, Faisal Dankishiya, Adamu Abdullahi Atterwahmie, Abubakar Shehu Muhammad, John Ekweani, Emily Nzeribe, Alfred Osoti, George Gwako, Jenipher Okore, Amani Kikula, Emmy Metta, Ard Mwampashi, Cherrie Evans, Kristie-Marie Mammoliti, Adam Devall, Arri Coomarasamy, Ioannis Gallos, Olufemi T. Oladapo, Meghan A. Bohren, Fabiana Lorencatto

**Affiliations:** a Centre for Behaviour Change, University College London, London, United Kingdom.; b Gender and Women’s Health Unit, Nossal Institute for Global Health, School of Population and Global Health, University of Melbourne, Melbourne, Australia.; c Department of Obstetrics, Gynaecology, and Reproductive Sciences, School of Medicine, University of California, San Francisco, CA, USA.; d Africa Center of Excellence for Population Health and Policy, Bayero University, Kano, Nigeria.; e Department of Obstetrics and Gynaecology, University of Nairobi, Nairobi, Kenya.; f Department of Obstetrics and Gynecology, Muhimbili University of Health and Allied Sciences, Dar es Salaam, Tanzania.; g Effective Care Research Unit, University of the Witwatersrand and Walter Sisulu University, Johannesburg, South Africa.; h Department of Obstetrics and Gynecology, University of Botswana, Gaborone, Botswana.; i KwaZulu-Natal Department of Health; and Department of Obstetrics and Gynaecology, School of Clinical Medicine, College of Health Sciences, University of KwaZulu-Natal, Durban, South Africa.; j Department of Obstetrics and Gynaecology, University of Cape Town, Cape Town, South Africa.; k Federal Medical Center, Nguru, Nigeria.; l Abubakar Tafawa Belawa University Teaching Hospital, Bauch, Nigeria.; m Federal Medical Center, Abuja, Nigeria.; n Federal Medical Center, Owerri, Nigeria.; o Department of Public Health, Institute of Tropical Medicine, Antwerp, Belgium.; p Global Health Institute, Faculty of Medicine and Health Sciences, University of Antwerp, Antwerp, Belgium.; q Department of Behavioural Sciences, Muhimbili University of Health and Allied Sciences, Dar es Salaam, Tanzania.; r Maternal and Newborn Health Unit, Technical Leadership and Innovation, Jhpiego, Baltimore, MD, USA.; s WHO Collaborating Centre on Global Women’s Health, Institute of Metabolism and Systems Research, College of Medical and Dental Sciences, University of Birmingham, Birmingham, United Kingdom.; t UNDP/UNFPA/UNICEF/WHO/World Bank Special Programme of Research, Development and Research Training in Human Reproduction, Department of Sexual and Reproductive Health and Research, World Health Organization, Geneva, Switzerland.

## Abstract

**Introduction:**

Postpartum hemorrhage (PPH) remains the leading cause of maternal mortality. A new clinical intervention (E-MOTIVE) holds the potential to improve early PPH detection and management. We aimed to develop and pilot implementation strategies to support uptake of this intervention in Kenya, Nigeria, South Africa, and Tanzania.

**Methods:**

Implementation strategy development: We triangulated findings from qualitative interviews, surveys and a qualitative evidence synthesis to identify current PPH care practices and influences on future intervention implementation. We mapped influences using implementation science frameworks to identify candidate implementation strategies before presenting these at stakeholder consultation and design workshops to discuss feasibility, acceptability, and local adaptations. Piloting: The intervention and implementation strategies were piloted in 12 health facilities (3 per country) over 3 months. Interviews (n=58), case report forms (n=1,269), and direct observations (18 vaginal births, 7 PPHs) were used to assess feasibility, acceptability, and fidelity.

**Results:**

Implementation strategy development: Key influences included shortages of drugs, supplies, and staff, limited in-service training, and perceived benefits of the intervention (e.g., more accurate PPH detection and reduced PPH mortality). Proposed implementation strategies included a PPH trolley, on-site simulation-based training, champions, and audit and feedback. Country-specific adaptations included merging the E-MOTIVE intervention with national maternal health trainings, adapting local PPH protocols, and PPH trollies depending on staff needs. Piloting: Intervention and implementation strategy fidelity differed within and across countries. Calibrated drapes resulted in earlier and more accurate PPH detection but were not consistently used at the start. Implementation strategies were feasible to deliver; however, some instances of limited use were observed (e.g., PPH trolley and skills practice after training).

**Conclusion:**

Systematic intervention development, piloting, and process evaluation helped identify initial challenges related to intervention fidelity, which were addressed ahead of a larger-scale effectiveness evaluation. This has helped maximize the internal validity of the trial.

## Abstract (precis)

We describe the systematic approach taken to identify areas of suboptimal postpartum hemorrhage detection and management to develop implementation strategies to support the delivery of the E-MOTIVE intervention in 4 countries.

## Teaser

**Key Findings:**

- Before introducing a new clinical intervention to improve the early detection of postpartum hemorrhage, we used multiple data collection methods (interviews, surveys, observations, and stakeholder workshops) to have a broad and in-depth understanding of key implementation issues, which included staff and drug shortages, limited in-service training, and different attitudes of staff toward adopting a new clinical intervention despite its perceived benefits.
- Conducting a pilot study with a process evaluation identified required modifications to implementation strategies before a larger randomized trial.
- Delivery of a new clinical care bundle and supporting implementation strategies for earlier postpartum hemorrhage detection and management were largely acceptable and feasible.
- However, fidelity was initially more challenging for staff when it required significant changes to existing practices for detection and management of postpartum hemorrhage.

**Key Implications:**

- Findings from the formative research helped to identify potential implementation issues and enabled refinement and increased preparedness before a larger-scale randomized trial.
- Collaborations across research disciplines and with health care providers enhance the development, design, and dissemination of new clinical interventions and their implementation strategies.

## INTRODUCTION

Postpartum hemorrhage (PPH) remains the leading cause of maternal mortality,^1^ accounting for up to 27% of all maternal deaths, with disproportionate burden in low- and middle-income countries (LMICs).^2^ PPH is defined as blood loss of 500 ml or more, and severe PPH as blood loss of 1,000 ml or more after birth.^3^ Etiologies include uterine atony (the uterus not contracting after birth), retained placental tissue, genital tract trauma, and coagulopathies.^4^ Severe morbidity and mortality associated with PPH are preventable when detected early and treated by prompt evidence-based clinical interventions, as recommended by World Health Organization (WHO) guidelines.^4^^,^^5^ While trends in maternal mortality rates suggest a global decrease in PPH between 2000 and 2017^3^ and stagnation between 2016 to 2020,^2^ delays in PPH detection, ineffective management, or a combination continue to be substantial challenges.^6^ Existing research has identified a range of factors influencing PPH detection and management in LMICs, including limited knowledge about current clinical guidelines and few training opportunities for health workers.^7^^–^^9^ Other contextual barriers include shortages of staff, drugs, and equipment and inadequate referral pathways, combined with poor transportation infrastructure.^7^^–^^9^

**FIGURE 1:**
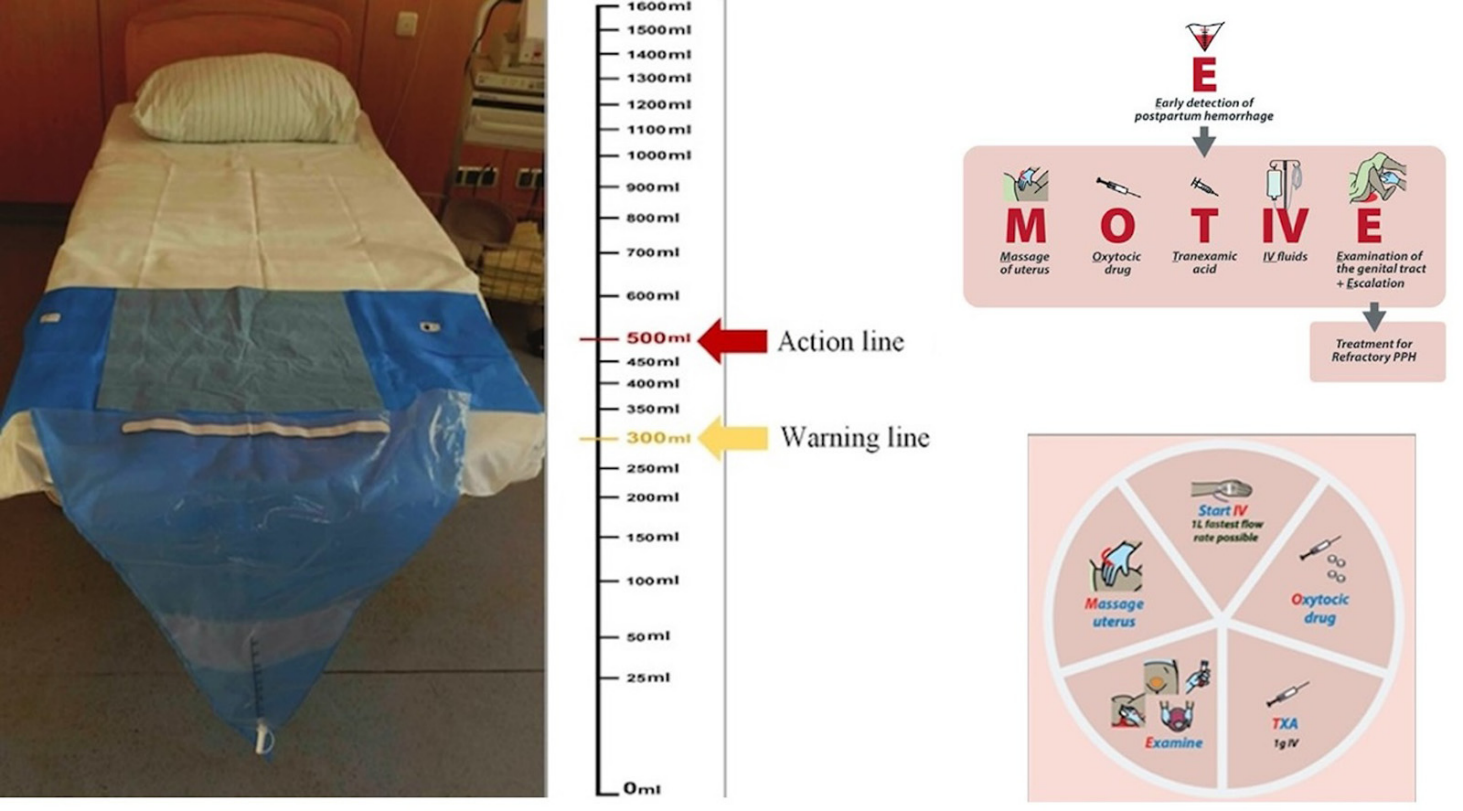
The E-MOTIVE Intervention Early Detection Calibrated Blood Collection Drape With WHO First-Response Treatment Bundle Abbreviations: E-MOTIVE, early detection of postpartum hemorrhage using a calibrated drape, massage of uterus, oxytocic drugs administration, tranexamic acid administration, intravenous fluids administration, examination for identifying and managing the source of bleeding, and escalation to more advanced care, if bleeding continues despite treatment; IV, intravenous; PPH, postpartum hemorrhage; TXA, tranexamic acid; WHO, World Health Organization.

Building on these “missed opportunities” to improve PPH detection and management and increase adherence to evidence-based recommendations for PPH treatment,^4^^,^^10^^,^ a new PPH first response clinical care bundle was proposed by a WHO Technical Consultation.^11^ A clinical care bundle is a set of evidence-based interventions designed to be administered together to improve adherence to evidence-based guidelines.^12^ The University of Birmingham, along with global collaborators, researchers, clinicians, and project and program developers, added an early detection component to the bundle, resulting in the E-MOTIVE intervention (Figure 1).^13^ E-MOTIVE stands for early detection of PPH using a calibrated drape, followed rapidly by the MOTIVE bundle (in no particular order): massage of uterus, oxytocic drugs administration, tranexamic acid administration, intravenous (IV) fluids administration, examination for identifying and managing the source of bleeding, and escalation to more advanced care, if bleeding continues despite treatment.^11^^,^^13^

The introduction of health care innovations, including care bundles, requires health workers to change their clinical practice behaviors by either modifying current practice, adopting a new practice, or discontinuing a previous practice. Existing evidence reports a wide range of barriers and enablers influencing clinical care bundle uptake.^14^^,^^15^ Previous research identified likely barriers and enablers of recommended PPH care: delayed detection, a lack of knowledge and training in PPH management, and shortages of staff and drugs.^7^^,^^11^ Therefore, implementing the new E-MOTIVE intervention is likely to necessitate implementation strategies to encourage uptake and sustainability.

Implementing the new E-MOTIVE intervention is likely to necessitate implementation strategies to encourage uptake and sustainability.

Theories and frameworks from behavioral and implementation sciences can aid in identifying barriers and enablers of behaviors that need to be targeted in the development of implementation strategies.^16^^,^^17^ Theory-based interventions are more likely to be effective or provide explanations for why an intervention has not worked.^18^ Therefore, applying behavioral change theory can facilitate exploration of the influences on current PPH treatment practices and “what it would take” to implement the new E-MOTIVE intervention in practice. However, to date, there has been limited theory-based research exploring factors influencing PPH detection and management in LMICs. This study applies a recognized approach to developing and evaluating behavior change interventions, the Behavior Change Wheel (BCW), which is a synthesis of 19 theoretical frameworks to guide the development of implementation strategies.^19^ The BCW uses the Theoretical Domains Framework (TDF)^20^^,^^21^ and the COM-B model (Capability, Opportunity, Motivation–Behavior) to guide intervention development by first conducting a behavioral analysis to identify what needs to change and then mapping to BCW intervention types (e.g., training, modeling, physical restructuring) to bring about the desired change.^19^

This study is part of the larger E-MOTIVE research program (ClinicalTrials.gov: NCT04341662), which aimed to design, develop, and pilot theory-based implementation strategies to support the E-MOTIVE intervention before conducting a large-scale cluster-randomized trial with process and economic evaluations in 4 countries (Kenya, Nigeria, South Africa, and Tanzania).^22^ This research comprised 3 workstreams that map onto the Medical Research Council framework for complex intervention development and evaluation.^23^ As part of the E-MOTIVE research program, we have already conducted mixed-methods formative research to understand factors influencing pre-intervention PPH detection and management and to prospectively identify health workers’ perceived barriers and enablers to implementing the E-MOTIVE intervention.^24^^,^^25^ The cluster-randomized clinical trial has since been completed and reported elsewhere.^26^ The trial results demonstrated a 60% relative reduction in the primary composite adverse PPH outcome (severe PPH 1,000 ml or more, laparotomy for bleeding, or maternal death from bleeding; risk ratio, 0.40, 95% CI=0.32, 0.50).^26^ The parallel process evaluation has been conducted and will explore and report on the fidelity, acceptability, and feasibility of the E-MOTIVE intervention in the cluster-randomized trial context. This article specifically reports on the stage of research that was conducted before the trial, which involved triangulation of the formative research, stakeholder involvement, and then piloting and evaluating the E-MOTIVE intervention and implementation strategies in a limited number of facilities before the larger clinical trial to inform any necessary intervention refinement ahead of larger-scale evaluation.

## METHODS

### Design

**FIGURE 2:**
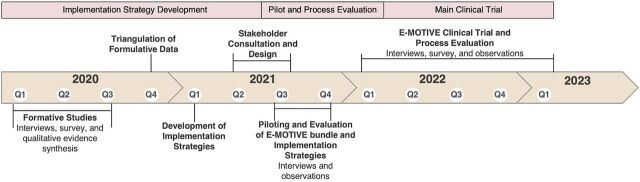
Timeline of E-MOTIVE Research Program Abbreviation: E-MOTIVE, early detection of postpartum hemorrhage using a calibrated drape, massage of uterus, oxytocic drugs administration, tranexamic acid administration, intravenous fluids administration, examination for identifying and managing the source of bleeding, and escalation to more advanced care, if bleeding continues despite treatment.

The study followed a 2-phase, 5-step systematic approach using behavioral and implementation science frameworks. A full protocol has been published.^22^ We report the methods in 2 phases: (1) implementation strategy development and (2) piloting and process evaluation of the E-MOTIVE intervention and implementation strategies. A mixed-methods approach involving qualitative interviews, surveys, observations, and stakeholder workshops was used in both phases. Figure 2 outlines the timeline of the phases and the steps taken in each phase. The research conducted in step 6 will be reported elsewhere.

### Phase 1: Implementation Strategy Development

#### Triangulating Data Sources

Triangulation data sources were qualitative interviews with health care professionals (n=45; 12 hospitals),^24^^,^^25^ a previously published qualitative study in Tanzania,^7^ an online survey with health care professionals (n=972; 91 hospitals), and a qualitative evidence synthesis (43 studies).^27^^,^^28^ Hospitals participating in the formative interview and survey studies met the inclusion criteria for the E-MOTIVE trial^26^ and were secondary-level hospitals in Kenya, Nigeria, South Africa, and Tanzania, had 1,000 to 5,000 vaginal births per year, and were able to provide comprehensive obstetrical care, with the ability to perform surgery for PPH. Within hospitals meeting these inclusion criteria, we used maximum variation sampling to recruit a diverse sample of hospitals based on size and geographical location. We also used maximum variation sampling within hospitals to recruit a diverse participant sample representing the different health care professional cadres who detect and manage PPH, including doctors, nurses, and midwives. The aim of triangulation was to integrate findings across these 3 formative research activities to arrive at a distilled set of key findings presenting current PPH practices and potential influences on the E-MOTIVE intervention and implementation strategies.^29^^,^^30^ First, the interviews, survey, and qualitative evidence synthesis findings were deductively mapped to the TDF.^20^^,^^21^ This integrates constructs from 33 behavior change theories into 14 domains representing the broad range of influences on behavior, including individual (i.e., knowledge, emotions, and perceived capability), sociocultural (e.g., social and professional role, and identity), and environmental (e.g., environmental context and resources) influences.^20^^,^^21^ TDF has been widely used to identify and synthesize influences on implementation and health care provider behavior.^31^ Following recommended triangulation methods,^29^^,^^30^ we created a matrix structured around findings from each data source and country. Five research team members independently rated each finding across data sources using convergence criteria^29^^,^^30^: “agreement” (i.e., finding is identified); “partial agreement” (i.e., partially covered finding); “disagreement” (i.e., opposed finding) and “not found” (i.e., finding is not present). This analysis was first conducted for Nigeria and then extended to Kenya, South Africa, and Tanzania. We then compared convergence and divergence of data across countries, resulting in key influences being grouped into main findings.

#### Designing Implementation Strategies

The next step was to generate recommendations for potential implementation strategies to address the key findings identified through triangulation. This step was guided by behavior and implementation science frameworks whereby TDF domains were mapped to the COM-B model and BCW.^19^ The research team consulted matrices pairing the 2 frameworks to identify potential intervention types,^32^^,^^33^ discussed how these could potentially be operationalized in the context of E-MOTIVE, and generated descriptions of candidate strategies, including diagrams and pictures.

#### Refining Proposed Implementation Strategies

We then hosted 2-day stakeholder consultation and design workshops separately for each country (4 workshops in total) to actively seek the knowledge, experiences, and perceptions of local stakeholders to refine proposed implementation strategies and to prospectively improve feasibility and acceptability. We invited a range of stakeholders, including senior management (head of obstetrics and matron-in-charge) and labor and delivery ward staff (consultant obstetricians, doctors, midwives, and nurses). All workshops were held virtually on Zoom due to COVID-19 restrictions in place at the time. During the workshops, the research team first presented the findings of the formative research and then described the E-MOTIVE intervention and implementation strategies. Then, we facilitated discussion about feasibility and acceptability, how best to design and deliver each strategy, and whether any adaptation to each country’s local context was needed. Feasibility was defined as the practicality of use, and acceptability was defined as “a multifaceted construct that reflects the extent to which people delivering or receiving a health care intervention consider it to be appropriate.”^34^ The workshops were audio-recorded and analyzed to identify necessary refinements and delivery preferences for countries and cadres, which were made ahead of piloting.

### Phase 2: Piloting and Evaluating the E-MOTIVE Intervention and Implementation Strategies

This phase aimed to field-test and pilot the E-MOTIVE intervention and implementation strategies, evaluate key implementation outcomes, and identify what needed to be refined and/or omitted. The pilot phase was conducted in 3 hospitals in each country (n=12) over a 3-month period (Clinicaltrials.gov: NCT04341662). The pilot aimed to replicate the cluster-randomized trial^26^ by purposively sampling hospitals to ensure representativeness of hospitals participating in the trial. Staff in participating hospitals were initially trained in E-MOTIVE by external trainers (Jhpiego), and the implementation strategies (e.g., champions and PPH trolleys/kits) were put into place. Hospitals received calibrated drapes during the pilot to enable early detection of PPH but also to pilot objective trial outcome measures (i.e., weighing drapes to measure blood loss). The weighing of drapes for trial outcome assessment was the responsibility of research midwives employed by the study at each site. Drugs were not supplied as part of the pilot.

Toward the end of the pilot period, we conducted a process evaluation consisting of direct clinical observations of women with vaginal birth and PPH and qualitative interviews. Country research teams also completed case report forms (CRFs) to collect data on the number of vaginal births, number of women with PPH, adherence to the MOTIVE bundle for women with PPH, and individual MOTIVE components delivered. Individual CRFs were collected for every woman giving birth at study hospitals. The CRFs were completed by research staff at each hospital and entered directly from the medical record to the REDCap electronic data capture system. Research staff at each hospital also collected drug stock-outs on a monthly facility-based CRF and entered this into the REDCap electronic data capture system. Hospitals taking part in the pilot met the inclusion criteria for the cluster-randomized trial but were excluded from participating in the trial to maintain internal validity and reduce the risk of contamination.

The key implementation outcomes evaluated were fidelity, feasibility, and acceptability. Fidelity was defined as the extent to which the E-MOTIVE intervention and implementation strategies were delivered as intended (i.e., adherence to the study protocol). Published criteria for interpreting fidelity suggest lower than 50% adherence represents “low” fidelity, 51%–79% “medium” fidelity, and 80%–100% “high” fidelity.^35^ We used a mixed-methods approach, comprising interviews with health workers; direct observations of health workers supporting women with vaginal birth and detecting and treating PPH; and CRFs of vaginal births, PPH cases, E-MOTIVE adherence, and drug stock-outs from patient and facility records to assess these implementation outcomes. Interviews assessed feasibility, including questions about the barriers and enablers to implementation using the TDF.^36^ To assess acceptability, interview questions were structured around the Theoretical Framework of Acceptability.^34^ Fidelity was self-reported in interviews, complemented through objective direct observations using a structured data collection form and woman-level data using the aforementioned CRFs. We used maximum variation sampling to recruit interview participants of different cadres, genders, and with varying years of experience. In-country research teams recruited interview participants, conducted the interviews, and completed the clinical observations over a 2-day period.

### Ethical Approval

This study was approved by the University of Birmingham (reference number: ERN_19-1557), the WHO Research Project Review Panel, and WHO Research Ethics Review Committee (reference number ERC.0003486) for the formative phase and the following relevant ethics and regulatory review committees in each country: University of Melbourne Medicine and Dentistry Human Ethics Sub-Committee (1956004) in Australia; National Health Research Ethics Committee of Nigeria (reference number: NHREC/01/01/2007)-07/04/2020) in Nigeria; University of Nairobi KNH-UON ERC (P25/01/2020) and the Pharmacy and Poisons Board PPB/ECCT/20/06/06/2020(116), National Commission for Science, Technology and Innovation Nacosti P/21/8437 in Kenya; Eastern Cape Department of Health (EC_202007_015), University of Cape Town Human Research Ethics Committee (reference number: 091/2020), Health Province of KwaZulu-Natal (reference number: KZ_202008_036), and University of the Witwatersrand Human Research Ethics Committee-Medical (reference number:M200241) in South Africa; and Muhimbili University of Health and Allied Sciences (reference number: DA.282/298/01.C/) and the National Institute for Medical Research (Reference number: NIMR/HQ/R.8a/Vol IX/3501) in Tanzania.

## RESULTS

We report the findings of the triangulation, mapping to implementation strategies, and stakeholder consultation and design workshops (phase 1), followed by the findings of the process evaluation of the pilot (phase 2).

### Phase 1: Development of Implementation Strategies

#### Triangulation

Table 1 shows the convergence of 11 key findings across data sources and countries (Supplement 1 shows the full set of findings). Across countries, findings show there was agreement across all data sources that most MOTIVE bundle components (uterine massage, oxytocic drugs, IV fluids, and examination/escalation) were already routinely used individually to treat PPH but not used in a bundled approach. Tranexamic acid, which has been recommended for PPH treatment,^10^ was not routinely used. There was agreement across all countries that introducing a new blood loss collection and measurement tool (i.e., calibrated drape) would need training, and there were some concerns about how this would replace existing blood loss estimation methods (e.g., visual estimation using blood-soaked linens or collecting blood in kidney dishes). However, generally, it was perceived that use of a calibrated drape would help detect PPH earlier. It was reported that the use of the MOTIVE bundle would require sufficient staff for teamwork and that the MOTIVE bundle would likely be effective at improving PPH treatment and reducing PPH morbidities and mortalities. There was also agreement that implementing the MOTIVE bundle would depend on having consistent stocks of drugs and equipment, as well as adequate staffing, as it was perceived that 1 person alone would not be able to deliver the MOTIVE bundle.

**TABLE 1.** Overview of Key Findings About Future Uptake of a New Bundled Approach to PPH Detection and Management Across Data Sources and Countries

	Kenya		Nigeria		South Africa		Tanzania		QES
	Interviews	Survey	Interviews	Survey	Interviews	Survey	Interviews	Survey	
**PPH detection**									
Use of a new measurement tool such as the calibrated drape will require staff being trained in how to use it	Agree	Not found	Agree	Not found	Agree	Not found	Not found	Not found	Not found
The new measurement tool (calibrated drape) can accurately measure blood loss which consequently could result in more PPH being detected	Agree	Not found	Agree	Not found	Agree	Not found	Not found	Not found	Not found
Concerns about how the new measurement tool (calibrated drape) will fit in with current methods of collecting blood	Agree	Not found	Agree	Not found	Agree	Not found	Not found	Not found	Not found
**PPH treatment**									
All components of the care bundle are part of current PPH management except tranexamic acid which is not routinely administered	Agree	Partially Agree	Agree	Partially Agree	Agree	Partially Agree	Not found	Partially Agree	Not found
Training on using tranexamic acid is required particularly for midwives who currently may not administer IV medications	Agree	Partially Agree	Agree	Agree	Agree	Disagree	Not found	Partially Agree	Not found
Use of multiple interventions like in the PPH care bundle depends on having a large team available; one person cannot deliver the care bundle	Agree	Agree	Not found	Partially Agree	Agree	Partially Agree	Partially Agree	Partially Agree	Not found
Future uptake of a care bundle could be effective at changing how PPH is currently treated for vaginal births	Agree	Partially Agree	Agree	Partially Agree	Agree	Partially Agree	Not found	Agree	Not found
**Overall use of a “bundled” approach to PPH treatment**									
Uptake of multiple interventions when treating PPH (e.g., MOTIVE bundle) will be challenging for staff to implement in the future	Disagree	Partially Agree	Disagree	Partially Agree	Disagree	Partially Agree	Not found	Partially Agree	Not found
The uptake of MOTIVE bundle depends on the confidence of staff to deliver all components as a bundle, i.e., all at once or in quick succession	Not found	Not found	Partially Agree	Not found	Partially Agree	Not found	Not found	Not found	Not found
Uptake of MOTIVE bundle relies on having sufficient staff on wards and having a reliable supply and consistent stock of the necessary drugs and equipment	Agree	Agree	Agree	Agree	Agree	Agree	Agree	Agree	Agree
The MOTIVE bundle to detect and treat PPH is likely to improve current management of vaginal births and reduce PPH-related mortality	Agree	Agree	Agree	Agree	Agree	Agree	Not found	Agree	Not found

Abbreviations: MOTIVE, massage of uterus, oxytocic drugs administration, tranexamic acid administration, intravenous fluids administration, examination for identifying and managing the source of bleeding, and escalation to more advanced care, if bleeding continues despite treatment; PPH, postpartum hemorrhage; QES, qualitative evidence synthesis.

Across countries, findings show there was agreement across all data sources that most MOTIVE bundle components were already routinely used individually to treat PPH but not used in a bundled approach.

Across countries, some differences were noted around the division of PPH treatment by different cadres (Kenya and Nigeria), in the use of tranexamic acid to treat PPH (Nigeria and Tanzania), and availability of calibrated drapes to measure blood loss (Kenya, Nigeria, Tanzania, and to less extent South Africa). There were also differences in receiving in-service training and existing skillsets among health workers. In the qualitative evidence synthesis, the provision of in-service training was reported as unsatisfactory, whereas participants in all countries receiving regular training (except Nigeria) reported being appropriately skilled at detecting and managing PPH. For the MOTIVE components, areas of disagreement were noted between countries about midwives not being trained to administer some components of the bundle, such as tranexamic acid (Kenya and Nigeria). Interview participants in all countries said the MOTIVE bundle as described would not be challenging to implement, whereas surveyed participants who did not have the bundle explained to them said rapidly administering multiple interventions (proxy for the MOTIVE bundle) might be challenging.

#### Mapping to Implementation Strategies

A summary of the mapping of the triangulated findings to the BCW to identify the components of the proposed implementation strategies is presented in Table 2. The proposed implementation strategies represent those agreed with stakeholders during the stakeholder consultation workshops to be feasible to deliver within the scope and context of the E-MOTIVE trial. The E-MOTIVE implementation strategy comprised 4 components:

**TABLE 2.** Mapping of Triangulated Findings Using BCW to Identify Proposed Implementation Strategies for the E-MOTIVE Intervention

**Summary of Findings**	**COM-B**	**Intervention** **Types From BCW**	**Proposed Implementation Strategies for E-MOTIVE Trial Context**
Need for earlier PPH detection from accurate blood loss measurement	Capability(physical and psychological)	EducationTrainingEnablement	Training of new skills to use the calibrated drape: new blood loss collection and measurement tool to prompt and facilitate earlier detection.Training of core or new skills to deliver MOTIVE bundle including simulation practice drills.
Need for training on using the new calibrated drape			
Uncertainty about administering tranexamic acid when not trained to do so			
Using the intervention brings challenges for staff including new skills and confidence building to deliver intervention as intended			
Need for sufficient staff and adequate stocks of PPH drugs and equipment	Opportunity (social and environmental)	Environmental restructuringModelingEnablement	Introduce PPH trolley or carry case to organize PPH drugs and equipment in 1 place accompanied by a checklist detailing the medicines and equipment to be stocked (country and site specific).Team-based training with simulation practice and drills to encourage and enable better coordination, teamwork, and communication when managing a PPH.
Need for teamwork to implement intervention			
Intervention use will bring about positive changes to PPH treatment	Motivation (reflective)	EducationPersuasionModelingEnablement	Introduce audit and feedback to monitor MOTIVE bundle uptake.Introduce a person in leadership role to “champion” the E-MOTIVE intervention implementation.
Uncertainty about how using the calibrated drape will fit in with current methods for collecting blood			
Perceptions that intervention use can potentially reduce PPH mortality			

Abbreviations: BCW, Behavior Change Wheel; COM-B, capability, opportunity, motivation-behavior; E-MOTIVE, early detection of PPH using a calibrated drape, followed by massage of uterus, oxytocic drugs administration, tranexamic acid administration, intravenous fluids administration, examination for identifying and managing the source of bleeding, and escalation to more advanced care, if bleeding continues despite treatment; PPH, postpartum hemorrhage.

1. Simulation-based team-based training to develop facility trainers (delivered by Jhpiego) and implemented on-site by facility trainers with follow-up practice drills on the calibrated drape and MOTIVE bundle use to encourage and enable staff to develop the capability to work and communicate in a coordinated and efficient manner as a team when detecting and managing a PPH.
2. Introduction of 2 local champions (1 midwife and 1 doctor) to promote E-MOTIVE uptake and provide reminders, encouragement, and social support to staff implementing E-MOTIVE.
3. Availability of a PPH trolley or carry case with all necessary drugs and equipment.
4. Provision of audit and feedback to all staff.^25^

The champions and audit and feedback strategies provided opportunities to deliver nonmaterial incentives and social encouragement for implementing E-MOTIVE by recognizing and praising instances of good practice.

#### Stakeholder Consultation and Design Workshops

Four interdisciplinary workshops (1 per country) were facilitated with a total of 63 participants: 28 nurses and midwives, 13 doctors, 16 consultants/specialists, and 6 clinical management staff. Most participants across all countries agreed in principle that the E-MOTIVE intervention and implementation strategies were implementable in their contexts. However, there were some challenges identified around adopting new ways of working and uptake of some aspects of individual implementation strategies. Table 3 presents the E-MOTIVE intervention and implementation strategies, workshop findings, and country-specific adaptions. All stakeholder decisions are available in Supplement 2.

**TABLE 3.** Findings From Stakeholder Consultation and Design Workshops and Country-Specific Adaptations

**E-MOTIVE Intervention**	**Intervention/Implementation Strategy Summary**	**Workshop Findings**	**Country-Specific Adaptations**
PPH detection:Calibrated drape	A calibrated drape will be tied around the woman’s waist after the baby is born to collect blood loss for the first hour after birth; if bleeding continued beyond the first hour, blood loss will be collected for a second hour.The calibrated drape includes 2 trigger lines at 300 ml and 500 ml of blood loss.Monitor vital signs (blood pressure and pulse rate), do clinical observations (uterine tone and vaginal blood flow) and take readings of calibration lines every 15 minutes and document it in patient notes.	Agreement on how to respond when blood loss reaches 300 ml (i.e., be alert to possible PPH) and at 500 ml (i.e., trigger MOTIVE bundle). Calibration lines were perceived as useful. However, some health workers may diagnose a PPH at 300 ml when there are poor vital signs, so did not want to wait until 500 ml to act. Agreement on blood loss being recorded in patient notes every 15 minutes. Vital signs taken every 15 minutes and recorded in patient notes.	Limited scope to change 300 ml and 500 ml trigger lines because it is fixed by trial protocol.As agreed, what clinical actions should occur at specific volumes of blood loss:For Nigeria, Tanzania, and Kenya, the criteria for triggering the bundle were: Clinical diagnosis of PPH, as per usual practice. ≥500 ml blood loss in the drape. ≥300–500 ml blood loss + an abnormal vital sign or observation. South Africa adopted criteria (1) and (2) above, but not (3).As agreed, develop a blood loss monitoring tool to support reading of calibration line and recording.
PPH treatment:MOTIVE bundle	Give all treatments in quick succession described as a “bundled approach” to managing PPH	Local protocols do not always allow midwives to administer tranexamic acid. Tranexamic acid is either only given by doctors or by midwives under a doctor’s prescription. At some sites, only doctors would do an internal vaginal examination.	Limited scope to change the bundle components because it is fixed by WHO recommendations^4^As needed, adapt existing local protocols so midwives can administer tranexamic acid.Address skillsets in training as required (see E-MOTIVE training below).
**Implementation Strategy**			
E-MOTIVE Training	Training of on-site trainers provided by Jhpiego^a^ On-site trainers deliver a facility-based, hands-on approach of training to all nurses, midwives, and doctors working on maternity wards including follow-up (e.g., skills practice simulation drills).	Limited consensus on the frequency of initial training of staff by on-site trainer. Concerns about holding follow-up practice simulation drill on a weekly basis, especially at sites where there are few or staff shortages.	Merge E-MOTIVE training with Essential Steps in the Managing Obstetric Emergencies^b^ training to avoid any conflicts about best PPH practice (in South Africa). Adjust frequency of practice simulation drills (n=8) depending on the size of the workforce (in Nigeria, Tanzania). More training on tranexamic acid and internal examination specifically for midwives.
PPH trolley or carry case	PPH trolley or carry case including a content checklist both to be provided by the trial program	Agreement about having a PPH trolley in the labor wards. Having a PPH trolley may be too large for some sites, therefore a smaller carry case was preferred. Some sites already have drugs and equipment checklist which could be used instead of an additional checklist which could increase paperwork workload of staff.	Sites to select the type of PPH kit, e.g., carry case in smaller wards (in Kenya, Nigeria and Tanzania), PPH box/ compartment as part of existing obstetric emergency trolley (in South Africa). Store oxytocin and other PPH drugs requiring cold chain in fridge on labor and delivery ward. Other drugs can be stored in the PPH trolley/carry case. Adapt existing stock checklist and assign to check and restock.
E-MOTIVE champion	Two staff per hospital of different clinical cadres (midwife, doctor) to lead and promote implementation of E-MOTIVE intervention and implementation strategies.	Agreement that 2 champions of different clinical roles should be implemented. More clarity on the remit of a champion and how it fits in with other competing duties and if champions should be remunerated.	Produce a champion handbook (all countries). Provide a platform for champions to virtually meet to give support and to share successes and challenges. None of the champions will be remunerated (all countries).
Audit and feedback	On-site staff to document drugs used for PPH and stock-outs of PPH drugs. Trial program to share number of vaginal births, number of PPH, % of PPH cases given oxytocin and tranexamic acid using dashboard for use with all on-site staff.	Agreement about having audit and feedback. Believed that the draft audit and feedback dashboard was difficult to interpret. All staff on maternity unit to receive feedback.	Develop a simpler, less detailed format for sites and add preferred comparators (i.e., include all study sites in a country).

Abbreviations: E-MOTIVE, early detection of postpartum hemorrhage using a calibrated drape; massage of uterus, oxytocic drugs administration, tranexamic acid administration, intravenous fluids administration, examination for identifying and managing the source of bleeding, and escalation to more advanced care, if bleeding continues despite treatment; PPH, postpartum hemorrhage; WHO, World Health Organization.

^a^ Jhpiego is a nonprofit organization for international health affiliated with Johns Hopkins University (https://www.jhpiego.org/).

^b^ Essential Steps in the Management of Obstetric Emergencies is a skills and drills program for all maternity staff developed in South Africa.

In summary, the 300 ml and 500 ml trigger lines on the calibrated drape were acceptable, and the clinical responses for each trigger line were reported as practical and feasible by all countries. The concept of the MOTIVE bundle was acceptable, although local adaptations were required where there was no protocol for midwives to administer tranexamic acid because midwives do not usually have prescribing or administrating authority for IV medications. The education and training strategies were both acceptable if merged with existing training (e.g., Essential Steps in the Managing Obstetric Emergencies in South Africa).^37^^,^^38^ Some adaptations were likely; for example, having as many as 8 different follow-up practice drills could be burdensome for sites with shortages of staff (Nigeria and Tanzania). Having a PPH trolley was acceptable in all countries except for storing oxytocin, which requires refrigeration. A PPH carry case would be more feasible for smaller labor wards where space was limited, and in South Africa, the PPH carry case replaced the existing PPH box. Also, participants reported that it was more practical to use existing drug and equipment checklists instead of developing new trolley-specific checklists and for each site to assign 1 person to check and restock after each shift rather than collective responsibility. Introducing a champion was acceptable, provided there was a clear remit of roles and responsibilities (e.g., champion handbook). The audit and feedback approach needed simplification because the dashboard format proposed at the workshops was difficult to understand and interpret; this was agreed upon by all countries.

### Phase 2: Piloting and Process Evaluation

Table 4 presents the pilot results per country. In the pilot, there were a total of 10,052 vaginal births that resulted in 1,269 PPHs. Across all countries, the implementation of all MOTIVE components for women with PPH (as recorded in the CRFs) was 41.1% (522/1269), representing low fidelity. However, there was substantial variation in fidelity across countries: 10.7% (55/512; low fidelity) in South Africa, 57.3% (215/375; medium fidelity) in Nigeria, 62.1% (187/301, medium fidelity) in Kenya, and 80.2% (65/81, high fidelity) in Tanzania.

**TABLE 4.** Pilot Results by Country

	**Kenya**	**Nigeria**	**South Africa**	**Tanzania**	**Total**
Vaginal births,^a^^,^^b^ no.	2,446	2,613	2,856	1,669	9,584
PPH cases,^b^ no.	301	375	512	81	1,269
Rate of PPH cases (%)	301/2,446 (12.3)	375/2,613 (14.4)	512/2,856 (17.9)	81/1,669 (4.9)	1,269/9,584 (13.2)
Adherence of MOTIVE,^b^ no. (%)	187 (62.1)	215 (57.3)	55 (10.7)	65 (80.2)	522 (41.1)
Massage	213 (70.8)	337 (89.9)	278 (54.3)	77 (95.1)	905 (71.3)
Oxytocin	232 (77.1)	336 (89.6)	312 (60.9)	81 (100)	961 (75.7)
Tranexamic acid	218 (72.4)	293 (78.1)	224 (43.8)^c^	71 (87.7)	806 (63.5)
IV fluids	228 (75.7)	342 (91.2)	304 (59.4)	78 (96.3)	952 (75.0)
Examination	213 (70.8)	254 (67.7)	64 (12.5)	69 (85.2)	600 (47.3)
Availability of drugs,^d^ %					
Oxytocin	100	100	100	100	100
Tranexamic acid	100	98.4	94.4	99.6	98.1

Abbreviations: IV, intravenous; MOTIVE; massage of uterus, oxytocic drugs administration, tranexamic acid administration, intravenous fluids administration, examination for identifying and managing the source of bleeding, and escalation to more advanced care, if bleeding continues despite treatment; PPH, postpartum hemorrhage.

^a^ Only births with source-verified blood loss data.

^b^ From individual woman’s case report form.

^c^ There may have been underreporting of tranexamic acid due to confusion about naming; tranexamic acid is known as Cyclokapron in South Africa.

^d^ From monthly facility-based case report forms.

There was substantial variation in fidelity across countries, ranging from 10.7% in South Africa to 80.2% in Tanzania.

Process evaluation interviews were conducted with 58 health workers at the pilot study hospitals (Table 5). Eighteen women with vaginal birth and 7 women with PPH were observed at each of the 8 hospitals (Kenya=2; Nigeria=3 and South Africa=3).

**TABLE 5.** Characteristics of Interview Participants in Pilot Phase Process Evaluation

**Characteristics**	**Kenya (n=15)**	**Nigeria (n=15)**	**South Africa (n=14)**	**Tanzania (n=14)**	**Total (n=58)**
Gender					
Female	8	8	11	7	34
Male	7	7	3	7	24
Profession					
Doctor	5	8	1	3	17
Midwife or nurse	6	5	9	11	31
Research nurse	3	0	0	0	3
Clinical administrative staff	1	2	4	0	7
Overall years of experience					
Less than 1 year	3	0	0	0	3
1–4 years	5	0	2	6	13
5–9 years	6	5	6	5	22
More than 10 years	1	10	6	3	20

Table 6 summarizes the interview and observation findings according to the implementation outcomes of interest: fidelity, acceptability, and feasibility, starting with the E-MOTIVE intervention and then each implementation strategy in turn. Supplement 3 presents a full description of identified TDF domain barriers and enablers influencing the feasibility and acceptability of the E-MOTIVE intervention and implementation strategies.

**TABLE 6.** Overview of Implementation Outcomes Across Observations, Interviews, Audit of Drug Use and Assessment of Implementation Outcomes

**Implementation Outcome**	**Observations**^a^ **(18 observations of vaginal birth, 7 PPHs)**	**Interviews (n=58)**	**Overall Assessment of Implementation Outcomes** ^b^
**Fidelity**			
Calibrated drape	Time of placing the calibrated drape: After birth and before oxytocin for PPH prevention (n=12/18); before birth with funnel rolled up (n=5/18), (1 calibrated drape in South Africa was placed after birth and after oxytocin for PPH prevention). Who placed the drape: midwife conducting the birth (n=11/18) includes 1 student midwife, E-MOTIVE research midwife (n=7/18). The calibrated drape was placed by the E-MOTIVE research midwife in Kenya n=2/7 and Nigeria n=5/7. Checking of blood loss calibration lines: Read for 14/18 births, although position of drape varied (e.g., hanging over bed or flat on bed). Blood swept into funnel of drape: Kenya (n=2/7), Nigeria (n=7/7), South Africa (n=4/4). Blood swept from under the woman: Kenya (n=2/7 drape removal, n=5/7 not done). Nigeria (n=2/7 after birth, n=2/7 after placenta delivery n=1/7 drape removal, n=2/7 not reported). South Africa (n=1/4 after examination and suturing, n=1/4 after placenta and suturing, n=1/4 drape removal, n=1/4 once without timing). Time in place: Kenya (median: 34 min, range: 8–52 min); Nigeria (median: 63 min, range: 22–94 min); South Africa (median: 65 min, range: 57–74 min). Vital signs measured while drape on: Kenya (n=1/7), Nigeria (n=2/7), South Africa (n=7/7).	All participants reported always using the calibrated drape to detect PPH. (Noted: discrepancy between self-reported and observed practice).	Use of drape: Medium to high fidelity Placing of drape by regular staff not employed by E-MOTIVE: Low to medium fidelity
MOTIVE components	MOTIVE triggered at: blood loss ≥ 500 ml: n=4/7; ≥300 ml + abnormal clinical signs n=1/7; placenta took 15 minutes to deliver n=1/7; not recorded n=1/7. Time between birth and triggering MOTIVE: median=12 minutes (range 5–30 minutes). Administering of MOTIVE components: Massage n=7/7. Oxytocin infusion n=7/7. Other uterotonics (misoprostol n=4/7). Tranexamic acid n=7/7. IV fluid n=7/7. Facility-based CRFs (% increase in brackets: difference between baseline and pilot).^c^ Use of oxytocin (%). Kenya 83%, (+14%); Nigeria 95%, (+30%); South Africa 80%, (+33%); Tanzania 100%, (0%). Overall increase, 34%. Use of tranexamic acid. Kenya 83%, (+47%); Nigeria 90%, (+86%); South Africa 68% (+43%); Tanzania 100%, (+50%). Overall increase, 59%.	All participants said, they administered all MOTIVE components of the bundle in quick succession to manage PPH. Participants said, they did not wait to see if one treatment worked before giving another treatment.	Use of MOTIVE: High fidelity
Training	Dedicated space for skills sessions and PPH simulation. 22 simulations took place across countries. Staff (typically nurses and midwives) participated in 2–7 simulation sessions.	Across all countries, all participants reported receiving initial training by an on-site trainer. There were mixed responses about attending follow-up skills sessions and PPH simulation. Across all countries some participants said they have not taken part in any practice drill sessions (n=19).	Initial training: High fidelityFollow-up skills sessions and PPH simulation:Low to mediumfidelity
PPH trolley/carry case	PPH trolley taken to bedside n=1/7. All sites had an accessible PPH trolley which was checked either daily, weekly, or at beginning/end of shift and regularly restocked. Most likely checked by an E-MOTIVE research midwife instead of on-site staff. Substantial variation in the supplies kept in the PPH trolley especially in Kenya and Nigeria. CRADLE devices (to take vital signs) supplied by E-MOTIVE project were often missing. All countries kept oxytocin in fridges (as per local protocol).	All participants said there was a PPH trolley available on the labor ward; however, some participants said it was not always used.	Availability of trolley:High fidelityUse of trolley:Low fidelity
Champions	NA	Number of participants reporting champions at site and number of champions in situ (if known) were: Kenya (n=13); 1–3 champions/sites (doctors and nurses). Nigeria (n=12); 2 champions/site (doctors and nurses). South Africa (n=9): 1–2 champions /site (doctors and nurses). Tanzania (n=10); 1–2 champions/site (doctors and nurses).	Champions in place:High fidelity
Audit and feedback	NA	Participants, who were aware of audit and feedback (n=12/58). Not all participants were receiving audit/feedback (n=8/58); none in South Africa. Participants reported, consultants and doctors are more likely to receive feedback (n=5/58): in Nigeria and South Africa. Format is verbal and/or graphs (n=5), in Kenya and Tanzania. E-MOTIVE staff (i.e., research midwife, champion, or on-site trainer) deliver audit/feedback to staff (n=5) in Kenya and Tanzania.	Engagement with audit and feedback:Low fidelity
**Acceptability**			
Calibrated Drape and MOTIVE components	NA	Across all countries, participants were satisfied with using the calibrated drape to detect PPH and using the MOTIVE components to treat PPH.	High acceptability
Training, PPH trolley/carry case, champions and audit and feedback	NA	Across all countries, participants said the implementation strategies offered appropriate support and encouragement to improve uptake of the E-MOTIVE intervention and implementation strategies at their site.	High acceptability
**Feasibility**			
E-MOTIVE Intervention and implementation strategies	Practical use of the E-MOTIVE intervention, attending the training by nursing staff and using PPH trolley/carry case was demonstrated at all sites.	Across all countries, participants reported it was feasible to deliver the bundle and engage with the implementation strategies.	High feasibility

Abbreviations: CRF, case report form; NA, not assessed via observations; E-MOTIVE, early detection of postpartum hemorrhage, massage of uterus, oxytocic drugs administration, tranexamic acid administration, intravenous fluids administration, examination for identifying and managing the source of bleeding, and escalation to more advanced care, if bleeding continues despite treatment; PPH, postpartum hemorrhage.

^a^ No observation completed in Tanzania (fidelity of E-MOTIVE intervention use).

^b^ Assessment of implementation outcomes (fidelity, feasibility, acceptability) criteria: low=≤50%; medium=between 51%–79% and high ≥80%.

^c^ Data collected by University of Birmingham clinical trial team.

#### Fidelity of E-MOTIVE Intervention

In contrast to the CRF data on low adherence to the MOTIVE bundle components, overall fidelity of the calibrated drape and the MOTIVE bundle in self-reported interviews and observations was medium to high.

The drape was placed with the funnel open after the birth in 66.7% of the women (12/18) and before the birth with the funnel rolled up in 33.3% (6/18) by a midwife in 61.1% (11/18) or by an E-MOTIVE research midwife in 38.9% (7/18). Monitoring of blood loss was regularly checked in Kenya and South Africa but less often in Nigeria. Across countries, there was different timing of sweeping blood into the funnel of the drape (after birth, after placenta delivery, drape removal, and not done) across all countries. There were some inconsistencies in what materials were included in the drape funnel (blood only or blood and blood-soaked pads/gauze). There were substantial variations in how staff responded when the blood loss reached 300 ml (warning line) and 500 ml (action line) when compared with the E-MOTIVE protocol. For 4 of the 7 women with PPH (57.1%), the MOTIVE bundle was correctly triggered at 500 ml of blood loss. Once triggered, all MOTIVE components were administered (median time from starting first component to starting last component was 6 minutes [range 3–25 minutes]). These findings from the pilot sites were critical in refining the use of the calibrated drape before the E-MOTIVE intervention was introduced into the cluster-randomized trial.

In contrast, self-reported fidelity to the E-MOTIVE intervention was much higher, with health workers reporting always using the calibrated drape. At 300 ml blood loss, health workers said that they would call for help and prepare for a possible PPH. All health workers reported triggering PPH treatment at 500 ml blood loss and adhering to the bundled approach of giving all treatments rapidly. No health workers reported “waiting to see” if 1 component worked before giving the next component.

Therefore, from the direct observations and interviews, it is possible to infer fidelity to the calibrated drape use was medium to high, and it was feasible to administer the MOTIVE components within 15 minutes (per study protocol).

#### Acceptance and Factors Influencing Feasibility of E-MOTIVE Intervention

Overall, staff reported that the E-MOTIVE intervention was feasible despite some challenges. Across all countries, enablers to delivering the E-MOTIVE intervention fell within the TDF domains: environmental context and resources (having an adequate supply of calibrated drapes and sufficient staff on a shift to give treatments in quick succession), beliefs about capabilities (calibrated drapes allowed quicker and easier and more accurate blood loss measurement), beliefs about consequences (improved PPH detection), and social/professional role and identity (evidence to support midwives’ diagnosis versus a higher-level professional’s estimate of blood loss). Other enablers fell within the TDF domains: social influence (supportive managers to ensure adequate drug stocks), emotion (reduced stress), social professional role and identity (increased scope of midwives’ practice to use all bundle components), social influence (from working within a team who support each other and communicate better), and goals (importance of bundle to effectively reduce PPH-related maternal mortalities).

Barriers fell within the TDF domains: environmental context and resources (long-term supply of calibrated drapes and lack of available delivery beds to maintain calibrated drape use for 1 hour after birth, in contexts where a woman is moved from delivery room to postnatal room, lack of staff to give treatments in quick succession); knowledge (lack of understanding about bundled approach); skills (ability to place the calibrated drape and administer tranexamic acid); beliefs about capabilities (to correctly read the measurement lines); and social influences (women do not want the calibrated drape to be kept in place for up to 2 hours after the birth and/or are traumatized by having a PPH so refuse potential painful treatment). Other barriers were social/professional role and identity (midwives’ hesitancy to administer tranexamic acid, which requires new skills), memory, attention and decision-making (forgetting to give all treatments), and intentions (reluctant to adopt a care bundle when existing PPH treatment is considered effective).

#### Fidelity, Acceptance, and Factors Influencing Feasibility of Implementation Strategies

##### Training:

The fidelity of initial training was high; however, fidelity of follow-up practice sessions was low to moderate. This was evidenced by all staff receiving training from on-site trainers (from training logbook). Twenty-two simulation practice sessions were held to observe potential challenges or missed opportunities with drape use and MOTIVE administration. The main areas where the staff participating in practice sessions did not adhere to the training were not retrieving the PPH trolley (Kenya) and not rapidly running IV oxytocin (Kenya and Nigeria). In interviews, some health workers in South Africa remarked that simulations did not always reflect the realities of PPH treatment, for example, when there were competing emergencies on the ward.

Acceptability and feasibility of training were high, evidenced by staff welcoming more training on PPH. Enablers fell within the TDF domains: skills (training was adequate), beliefs about capabilities (building confidence) and memory, and attention and decision-making (remembering to deliver E-MOTIVE intervention). The TDF domain of environmental context and resources might be a barrier whereby the training of new staff makes time demands on the system to provide ongoing training on E-MOTIVE intervention use.

##### PPH trolley/carry case:

Although PPH trolleys were available, fidelity of their use was low. This was evidenced by all sites having easy access to a PPH “kit” of supplies, which was stored in either a trolley (Nigeria and Tanzania) or a carry case (Kenya and South Africa). In the interviews, health workers reported that the PPH trolley was used, and it was regularly checked and restocked on a daily, weekly, or after use basis. However, this contrasted with more objective observations, where it was observed that the PPH trolley was not frequently used (14.3%, 1/7).

Despite the low fidelity of the PPH trolley, acceptability of having a PPH trolley was reported as high. Reasons provided for not regularly using the PPH trolley included the trolley not being adequately stocked or beliefs that retrieving it was not a good use of health workers’ limited time. Additional influences on trolley use fit within the domains of environmental context and resources (keeping drugs and equipment together saves time unless space on the ward was limited and if the trolley was in good working order and there was sufficient staff to retrieve it), beliefs about consequences (improving PPH response time), knowledge (all ward staff knew where to find PPH items), beliefs about consequences (trolley could improve PPH treatment), and intention (no plans to use trolley because it was inadequately stocked).

##### Champions:

Fidelity, acceptance, and feasibility of having champions in place was high. This was evidenced by interview participants reporting having 1–3 champions per hospital. Some health workers were unsure about the clinical role of the champion and a few health workers were not aware of having a champion at their site. For those who were aware of the champions’ roles, having champions was considered appropriate. Interviewed participants highlighted some challenges with having champions, which included having limited capacity to carry out roles due to competing clinical tasks, appropriate division of responsibility and labor between 2 or more champions, and difficulties in champions working on different shifts from other staff, therefore, unavailable for some staff. However, champions were reported to give support, ensure supplies of drapes and stock of drugs, provide training to new staff, and manage any ill feelings or conflicts among staff. Enablers to engagement with champions fit within domains of social/professional role and identify (designated on-site staff fulfilling champion’s role), beliefs about consequences (helpful and supportive), and social influence (importance of having a “focal” person who can talk to staff). Barriers also fit within social influence (misunderstandings among staff and champions receiving financial incentives) and beliefs about capabilities (champions’ capacity due to other competing clinical responsibilities).

##### Audit and feedback:

Fidelity, acceptance, and feasibility of engaging in audit and feedback were low. This was evidenced by most interview participants being unaware of audit and feedback strategies, suggesting inconsistent fidelity across countries. Doctors were more likely to participate in feedback sessions than midwives (Kenya and South Africa). The TDF enablers for staff engaging with audit and feedback were beliefs about consequences (beneficial because it identified where improvements are needed), goals (important to set targets), and environmental content and resources (audit and feedback posters displayed in all hospitals). A key barrier related to beliefs about capabilities (perceived accuracy of the audit and feedback [i.e., staff felt actual drug use was higher or lower than expected] and drug use related to poor documentation skills) and memory, attention and decision-making (drugs were usually locked away [e.g., tranexamic acid] or if key holder going off duty forgets to leave keys for next shift).

## DISCUSSION

This article describes the systematic approach taken to identify areas of suboptimal PPH detection and management to develop implementation strategies to support delivery of the E-MOTIVE intervention. Using the BCW, the approach involved different country stakeholders and pilot testing of the implementation strategy to identify implementation issues. This approach provided an opportunity to identify what was working well and where to make further intervention modifications to improve implementation before the E-MOTIVE cluster-randomized trial.

This article describes the systematic approach taken to identify areas of suboptimal PPH detection and management to develop implementation strategies to support delivery of the E-MOTIVE intervention.

The pilot shows that the initial implementation of the E-MOTIVE intervention and implementation strategies in the pilot hospitals (which were distinct from the main E-MOTIVE trial hospitals) varied within hospitals and across countries. All components of the bundle were delivered; however, the new calibrated drape was not always consistently and appropriately used by staff, despite health workers acknowledging the reliability of the calibrated drape to measure blood loss quickly and accurately. This suggests switching to the calibrated drape requires time for staff to adapt to new ways of working and success depends not only on health workers understanding the benefits of accurate blood loss measurement. The overall use of oxytocin in women with PPH increased to 80% or higher in all countries except Tanzania, which was already 100% at baseline. Overall use of tranexamic acid in women with PPH also increased in all countries. While improvements in drug use were feasible, there was still an implementation gap in the fidelity of the MOTIVE components. Although fidelity varied across countries, given the relatively short duration of the pilot phase, the limited number of hospitals participating in the pilot, and relatively small number of women with PPH, it is not possible to draw firm conclusions about the relationship between extent of fidelity and PPH outcomes. This is something that will be explored as part of a larger-scale process evaluation conducted alongside the E-MOTVE cluster-randomized trial, with a larger number of participating facilities and over a longer time frame. The short time frame in the present pilot study may also, in part, explain the fact that drug stock-outs were not a reported barrier. The PPH trolley and kit introduced as part of the implementation strategy may have also facilitated the coordination and restocking of necessary medications and supplies.

While the piloting and process evaluation of the E-MOTIVE intervention and implementation strategies highlighted numerous enablers that indicate good feasibility and acceptability of E-MOTIVE, it also identified some barriers that highlight implications for refining E-MOTIVE ahead of the cluster-randomized trial. The pilot has shown that differences in E-MOTIVE intervention implementation could be explained by staff’s familiarity with the MOTIVE components from current PPH treatment practices compared to the more significant changes in PPH detection using the new calibrated drape. In response, the E-MOTIVE training and skill practices for using the calibrated drape needed to ensure the setting and equipment reflect the clinical reality. The interviews and observations showed the research midwives performing some aspect of E-MOTIVE implementation beyond their intended role of placing and weighing the calibrated drapes. Although this represents a deviation in fidelity or intended practice, it is potentially explained by the fact that research midwives may have felt ethical or moral obligations to assist in an emergency, such as PPH, even if support was not required. To try and address this ahead of the trial, Jhpiego (providers of train the trainer activity) updated the materials to stress that the research midwives’ role is to collect data, support training and practice, and help with managing the PPH trolley but not provide routine care. The intention that administration of the bundle should only be done by clinical staff was further emphasized in the E-MOTIVE training. The intervention was to be delivered by regular facility staff who conduct births, and nurses and midwives can implement the entire intervention when a doctor is not present. Alternatively, an additional recommendation for future studies or implementation would be to employ nonclinical research staff to assist with data collection and training to minimize the opportunity and likelihood of them supporting with delivery of clinical care and, in turn, impacting fidelity.

Some staff also reported knowledge barriers related to the drape and limited self-efficacy in interpreting calibration lines. Therefore, other changes included refining training materials to explain and reinforce the importance of calibrations to trigger the bundle, carrying out calibration reading practice, and making modifications to treatment provision (duration of massage and IV fluids with oxytocin to be infused from 1L to 500 ml). We found that the uptake of the supporting implementation strategies also varied across hospitals from all countries. While all health workers received training, there were differences in the organization of follow-up practice sessions, varying use of the PPH trolley, presence of and engagement with champions, and health workers’ receiving audit and feedback on MOTIVE bundle usage. In response, the number of follow-up practice sessions was reduced from 8 to 5, a new PPH trolley checklist was introduced to ensure supplies were restocked after use or daily (supported by research midwives if needed), and new training monitoring materials were introduced to ensure quality and consistency of implementation.

While initial uptake was not always as intended, the country partners worked to increase feasibility and, consequently, fidelity over the pilot period. For example, increased efforts were made to explain the role of the local champions (a new concept in some study sites) and ensure they were appointed as “the face” of any implementation efforts to help overcome resistance to change, promote consistent use of intervention, and promote uptake of implementation strategies. Specifically, efforts were made to encourage the uptake of audit and feedback, which was limited possibly because the existing culture was to only give feedback to senior staff, not all staff. Similar uptake issues were also identified for implementation strategies aimed at improving the clinical care bundle fidelity, such as “Sepsis Six.”^39^

This study highlights the importance of piloting interventions before large-scale evaluation of effectiveness and targeting different influencing factors on implementation. Our findings identify early challenges to implementing the E-MOTIVE intervention, particularly the introduction of the calibrated drape and some limited uptake of the PPH trolley/carry case, skills practice after training, champions, and audit and feedback implementation strategies. The generalizable lessons learned for use in other trials were to not rush from intervention development to trial, to pilot the intervention and implementation strategies, and to evaluate via a process evaluation first to identify areas for refinement ahead of larger-scale trials. This is important to help maximize intervention fidelity and, in turn, the likelihood of the trial being able to accurately evaluate if the intervention (E-MOTIVE + implementation strategies) is effective or not, reducing potential waste of resources, time, and efforts of testing at scale, if not working as optimally in the pilot. Our study highlighted a “know-do” implementation gap based on differences between real-world direct observations and reported use of E-MOTIVE and uptake of implementation strategies by interview participants. Therefore, when introducing new or modified ways of working, such as implementing a new care bundle and adopting implementation strategies, it is essential to be aware of potential issues with fidelity, acceptability, and feasibility.

Our study highlighted a “know-do” implementation gap based on differences between real-world direct observations and reported use of E-MOTIVE and uptake of implementation strategies by interview participants.

### Strengths and Limitations

The strengths of the study are that it was multidisciplinary collaborative research using rigorous data collection methods, and it adhered to Medical Research Council guidelines to involve stakeholders to achieve consensus through discussion of supporting implementation strategies. Other strengths included the use of behavior change theory to develop potentially more effective supporting implementation strategies, identification of any previously missed opportunities to improve PPH detection and management, and addressing any implementation issues of the PPH intervention and implementation strategies before the start of the cluster-randomized trial. Methodological limitations of the research were probable loss of interpersonal and group dynamics from using Zoom for workshops, inability to address some contextual issues (staff shortages and lack of beds), and the sustainably of stocking medicines and supplies. Direct observations limited to dayshifts may have missed better or worse performance. The repeated data collection may have resulted in participant fatigue if interviewed more than once and possible stress from being observed.

We acknowledge differences in fidelity were identified across the qualitative interviews, direct observations, and CRFs findings. Fidelity in self-reported interviews was high, in contrast to observations, which were medium, and CRFs, which were low. This difference can potentially be explained by the data collection biases associated with each method. Interviews are subject to social desirability and recruitment bias, and direct observations are subject to the Hawthorne effect,^40^ in which participants modify their behavior in response to their awareness of being observed. Moreover, low fidelity, as reported in the CRFs, may have been influenced by incomplete patient records. These discrepancies were anticipated in advance, hence, why a mixed methods approach was used to achieve a more balanced picture of implementation outcomes.

Additional limitations include, first, the focus of the E-MOTIVE program on PPH following births occurring in secondary-level hospitals meeting the trial criteria.^26^ The proportion of hospital-based births varies across countries, with births also occurring in lower-level hospitals and community settings (i.e., at home or in nonhospital health facilities). Therefore, the E-MOTIVE program of research and associated clinical and implementation interventions addresses a subset of PPH and associated morbidity and mortality, and consideration of adaptation of the drape, bundle, and implementation strategies for births in other settings is a next key step. The qualitative evidence synthesis^28^ that was conducted as part of the E-MOTIVE formative research to inform intervention development reports on the perceptions and experiences of the prevention, detection, and management of PPH following births in different settings, particularly from the perspective of women, community members, and traditional birth attendants. The qualitative evidence synthesis maps identified barriers and enablers in other settings to potential interventions and summarizes recommendations for implementation.^28^ Second, the reported triangulation of the findings from the formative research highlighted wider system-level barriers related to staffing levels and infrastructure (e.g., insufficient number of beds and electricity), which were beyond the scope of what was feasible and sustainable to address within the context of E-MOTIVE. Nonetheless, these highlight that ensuring adequate skilled workforce, infrastructure, supplies, and medications are important implementation considerations for policymakers looking to implement and sustain E-MOTIVE beyond the trial, should it be shown to be effective.

## CONCLUSION

From an implementation perspective, delivering the E-MOTIVE intervention and implementation strategies was mostly acceptable and feasible across 4 study countries during the pilot phase. The E-MOTIVE intervention, coupled with the described implementation strategies, has the potential to detect PPH earlier and more accurately and to improve PPH management, which may result in reduced PPH-related morbidity and mortality. Potential threats to fidelity and acceptability identified in the pilot phase can be addressed by refining the E-MOTIVE intervention and implementation strategies ahead of the cluster-randomized trial.

## Supplementary Material

GHSP-D-23-00387_supplements.pdf

## Notes

**Peer Reviewed**

**First Published Online:** September 12, 2024.
