# Supplementary material for: Development and Piloting of Implementation Strategies to Support Delivery of a Clinical Intervention for Postpartum Hemorrhage in Four sub-Saharan Africa Countries
Source: Glob Health Sci Pract. 2024 Oct 29;12(5):e2300387. doi: 10.9745/GHSP-D-23-00387 (PMC11521548; doi:10.9745/GHSP-D-23-00387)
Supplement: GHSP-D-23-00387_supplements.pdf [file GHSP-D-23-00387_supplements.pdf]

Supplement to: Forbes G, Akter S, Miller S, et al. Development and piloting of implementation strategies to support delivery of a clinical intervention for postpartum hemorrhage in four sub-Saharan Africa countries. *Glob Health Sci Pract*. 2024;12(5). https://doi.org/10.9745/GHSP-D-23-00387

SUPPLEMENT 1. Overview of the identified findings about current PPH practice and how they converge across data sources within study countries

FINDINGS	KENYA		NIGERIA		SOUTH AFRICA		TANZANIA		QES
	Interviews	Survey	Interviews	Survey	Interviews	Survey	Interviews	Survey	
**PPH Detection and Management**									
Overall, healthcare providers have a good understanding of how to define different types of PPH (i.e., mild, severe) and how to detect a PPH (i.e., skills, knowledge)	Agree	Agree	Agree	Agree	Partially Agree	Agree	Silence	Agree	Disagree
Overall, there is a good understanding of what constitutes appropriate clinical practice for management of PPH and when to escalate to refractory PPH management strategies	Partially Agree	Agree	Agree	Agree	Partially Agree	Agree	Silence	Agree	Partially Agree
PPH is detected using a combination of methods e.g., estimating blood loss, taking vital signs, using uterine tone and size	Agree	Agree	Agree	Agree	Agree	Agree	Silence	Agree	Agree
There is a lack of accurate and objective measurement of blood loss resulting in an over- or under-estimation of blood loss	Agree	Agree	Agree	Agree	Agree	Agree	Silence	Agree	Agree
Among healthcare providers there is confidence in detecting and managing a PPH after a vaginal delivery; the confidence of the individual and the and skillsets of the team can affect the quality of PPH care	Partially Agree	Agree	Partially Agree	Agree	Partially Agree	Agree	Partially Agree	Agree	Disagree
There are missed opportunities to improve PPH detection and management	Partially Agree	Partially Agree	Partially Agree	Agree	Partially Agree	Partially Agree	Partially Agree	Agree	Partially Agree
Tasks to manage a PPH are carried out by only certain clinical roles (e.g., only doctors administer tranexamic acid)	Partially Agree	Silence	Partially Agree	Silence	Disagree	Silence	Silence	Silence	Agree
Working as a team is highly valued, it is considered necessary to provide appropriate PPH care whereby staff have support and assistance readily available	Partially Agree	Agree	Partially Agree	Agree	Partially Agree	Agree	Agree	Agree	Agree
For teams to work effectively, there need to be good, regular and clear communication between the members of the team	Silence	Silence	Silence	Silence	Partially Agree	Silence	Silence	Silence	Agree
PPH care which has multi-discipline teams were more likely to facilitate changes in practice because it brings people together, builds local ownership, and promote accountability	Silence	Silence	Silence	Silence	Silence	Silence	Agree	Silence	Agree
PPH care benefits from good supervision and leadership which can provide support, inspire good teamwork and they can lead by example	Silence	Agree	Silence	Agree	Silence	Agree	Partially Agree	Agree	Agree

FINDINGS	KENYA		NIGERIA		SOUTH AFRICA		TANZANIA		QES
	Interviews	Survey	Interviews	Survey	Interviews	Survey	Interviews	Survey	
Disciplinary procedures are in place and staff can be disciplined because of malpractice	Partially Agree	Agree	Partially Agree	Agree	Agree	Partially Agree	Agree	Partially Agree	Agree
Healthcare providers can experience stress and anxiety resulting from unpredictability and fatality associated with detecting and managing PPH	Agree	Partially Agree	Agree	Partially Agree	Agree	Partially Agree	Agree	Partially Agree	Silence
PPH is a priority compared to other maternal health conditions	Partially Agree	Agree	Partially Agree	Partially Agree	Partially Agree	Agree	Silence	Agree	Agree
**PPH Training**									
Most healthcare providers have received some form of PPH training since medical/midwifery school in the last 2 years	Partially Agree	Agree	Partially Agree	Agree	Partially Agree	Agree	Silence	Partially Agree	Silence
The in-service training for healthcare workers is adequate; therefore, healthcare providers have the appropriate skills to detect and manage PPH	Agree	Agree	Partially Agree	Agree	Partially Agree	Agree	Silence	Partially Agree	Disagree
Most training is through presentations alone, with few healthcare professionals trained through case-based scenarios or simulation exercises	Silence	Agree	Silence	Agree	Silence	Agree	Silence	Partially Agree	Disagree
PPH training is more likely to be delivered by trainers based in the hospital than from district, regional, Ministry of Health or non-governmental organisation trainers	Silence	Partially Agree	Silence	Partially Agree	Silence	Partially Agree	Silence	Partially Agree	Silence
Additional PPH training is essential to help keep up to date with current practice and it an effective method to improve PPH detection and management	Agree	Agree	Agree	Agree	Agree	Agree	Agree	Agree	Agree
Training format is team-based, multi-disciplinary simulation training which can improve working in a team, improving confidence, motivation and reliability	Silence	Partially Agree	Silence	Partially Agree	Partially Agree	Partially Agree	Partially Agree	Partially Agree	Agree
**Improvement Initiatives**									
Maternity unit have protocols and guidance for PPH detection and management with guidelines on display in the labour and delivery rooms	Agree	Agree	Partially Agree	Agree	Partially Agree	Agree	Silence	Agree	Disagree
Guidelines for PPH are useful and relevant, and posters are effective methods in helping to improve PPH detection and management	Partially Agree	Agree	Partially Agree	Agree	Agree	Agree	Silence	Partially Agree	Silence

Supplement to: Forbes G, Akter S, Miller S, et al. Development and piloting of implementation strategies to support delivery of a clinical intervention for postpartum hemorrhage in four sub-Saharan Africa countries. *Glob Health Sci Pract*. 2024;12(5). https://doi.org/10.9745/GHSP-D-23-00387

FINDINGS	KENYA		NIGERIA		SOUTH AFRICA		TANZANIA		QES
	Interviews	Survey	Interviews	Survey	Interviews	Survey	Interviews	Survey	
Guidelines and protocols tailored to the needs of the local context are more helpful because some drug recommendations are not stocked or not routinely available	Agree	Silence	Agree	Silence	Silence	Silence	Silence	Silence	Silence
There is ongoing, sufficient monitoring and performance feedback in place at a facility level e.g., debriefing with colleagues by having a 'huddle' after an emergency or at the end of a shift quality of care received by woman, or at regular facility meetings	Agree	Agree	Agree	Partial Agree	Agree	Partially Agree	Silence	Partially Agree	Agree
Benefits of feedback from management staff is identifying areas of current practice requiring improvement	Agree	Agree	Agree	Agree	Agree	Agree	Agree	Agree	Silence
Feedback about care quality from women, relatives or the community helps to improve and change practice in hospitals	Silence	Silence	Agree	Silence	Silence	Silence	Silence	Silence	Silence
Disciplinary procedures are in place and staff can be disciplined because of malpractice	Partially Agree	Agree	Partially Agree	Partially Agree	Partially Agree	Agree	Silence	Agree	Silence
**Resources: Supply of drugs and equipment, and staffing levels**									
Tranexamic acid is not routinely stocked, or supplies are inconsistent, and some countries it must be procured by the woman or her relatives	Agree	Disagree	Agree	Partially Agree	Disagree	Disagree	Agree	Partially Agree	Agree
There is a not a reliable blood bank at the hospital, therefore there is a reliance on relatives to donate blood	Agree	Silence	Agree	Silence	Agree	Silence	Agree	Silence	Agree
Workforce related barriers to PPH care could be inadequate staffing levels especially during periods of high workloads and a high turnover of skilled healthcare providers	Partially Agree	Disagree	Agree	Disagree	Partially Agree	Disagree	Silence	Disagree	Agree
Supplies of tools to detect or to measure blood loss are readily available in the labour/delivery room	Disagree	Disagree	Disagree	Disagree	Silence	Disagree	Silence	Disagree	Disagree
Uterotonic drugs (i.e., oxytocin, misoprostol) and IV fluids, are available in hospitals	Agree	Agree	Agree	Agree	Agree	Agree	Agree	Agree	Partially Agree
**Environmental, Setting or Context Factors**									
All maternity facilities have varying capacity to cope with high volumes of referred women who are un-booked or emergency cases of PPH	Partially Agree	Silence	Silence	Silence	Silence	Silence	Silence	Silence	Silence

Supplement to: Forbes G, Akter S, Miller S, et al. Development and piloting of implementation strategies to support delivery of a clinical intervention for postpartum hemorrhage in four sub-Saharan Africa countries. *Glob Health Sci Pract*. 2024;12(5). https://doi.org/10.9745/GHSP-D-23-00387

**FINDINGS**	KENYA		NIGERIA		SOUTH AFRICA		TANZANIA		QES
	Interviews	Survey	Interviews	Survey	Interviews	Survey	Interviews	Survey	
PPH is a common occurrence at country level, but maternal mortality is low at my hospital	Partially Agree	Partially Agree	Agree	Partially Agree	Agree	Partially Agree	Agree	Agree	Silence
Concerns about women being referred to late by other facilities including a lack of required information about the referred woman and potential mismanagement of the PPH case by the referring facility	Agree	Silence	Partially Agree	Silence	Partially Agree	Silence	Partially Agree	Silence	Agree
Theatres are ready and surgical staff are available when required	Partially Agree	Agree	Partially Agree	Agree	Partially Agree	Agree	Silence	Agree	Silence

*Divergence from E-Motive Jhpiego training definitions when compared to Essential Steps in the Management of Obstetric Emergencies (ESMOE): A skills and-drills program for all maternity staff developed in South Africa

Convergence criteria for interviews and QES: ‘agreement’=finding is identified; ‘partial agreement’=partially covered finding; ‘disagreement’=opposed finding and ‘silence’= finding is not present

Agreement criteria for survey: Partial agreement is <75%, Agreement is ≥75%

Supplement to: Forbes G, Akter S, Miller S, et al. Development and piloting of implementation strategies to support delivery of a clinical intervention for postpartum hemorrhage in four sub-Saharan Africa countries. *Glob Health Sci Pract*. 2024;12(5). https://doi.org/10.9745/GHSP-D-23-00387

SUPPLEMENT 2. Key decisions from interdisciplinary workshops and at follow-up working group meetings

**Question**	**Kenya** (n=16)	**Nigeria** (n=17)	**South Africa** (n=14)	**Tanzania** (n=16)
**CALIBRATED DRAPE**				
**Usefulness of 300mls and 500mls trigger lines**<br><br>*Q1. To what extent would you find the 300ml and 500ml calibration lines useful?*	Warning for action at 300mls and not to wait until blood loss of 500mls<br><br>Yellow line (300mls) to indicate preparedness for a PPH<br><br>Prompts to order drugs, call for help and to get drugs	Both lines have a role in detecting a PPH.<br><br>300mls is an alert to act and 500mls triggers the bundle	Having a 300mls line complicates things; must not ignore vital signs [visualisation and vital signs should be complementary]<br><br>**Follow-up working group meeting**: Wait for the blood loss to reach 500mls then MOTIVE bundle is triggered	Both lines have a role in detecting a PPH. 300mls is an alert to act and 500mls triggers the bundle
**Documenting blood loss**<br><br>*Q2. How would you use this information (the amount of blood loss based on the calibration lines in the drape) in your practice?*	Document every 15 minutes, document more frequently for greater blood loss and<br><br>documenting blood loss is easy and beneficial	Apart from vital signs, also routinely write down every 15 minutes the blood loss from the drape calibration in patient notes/partograph (as per WHO recommendation)	Document every 15 minutes including cumulative volume, WC; document for 2-3 hours after delivery<br><br>**Follow-up working group meeting**: weighing the drape and recording it	Important to document every 15 minutes particularly by midwives
**Actions at 300mls**<br><br>*Q3. The yellow line is at 300mL, what actions do you suggest taking when the blood loss reaches that level?*	300mls has 3 roles: warning line, prepare, i.e., IV line/bring trolley kit near/ for anaemic mothers start at 300mls	Use it as a trigger to begin MOTIVE where one more variable is also abnormal such as vitals, uterine tone or size or heavy, rapid flow<br><br>Yellow line should be used as an alert line: 300mls + other parameters = action (i.e., not rely on blood loss only)	300mls is an alert line: a warning to be more vigilant and action to tell someone<br><br>KZN think yellow line is still normal blood loss	Both lines are important - 300mls will be treated as a warning line, preparing for treatment at the red line.
**Where to document blood loss**<br><br>*Q4. How would you want to document the information on blood loss provided by the drape?*	Record in patient notes (possible lack of space within notes for continuous monitoring); Provision in the partograph preferred;	A document (partograph) already exists and is used in the facilities to record vital signs; add timeline to existing partograph	Currently use maternity case records in all provinces	Record in documentation chart. Currently there would be a lack of space so consider a continuous sheet

Supplement to: Forbes G, Akter S, Miller S, et al. Development and piloting of implementation strategies to support delivery of a clinical intervention for postpartum hemorrhage in four sub-Saharan Africa countries. *Glob Health Sci Pract*. 2024;12(5). https://doi.org/10.9745/GHSP-D-23-00387

**Question**	**Kenya** (n=16)	**Nigeria** (n=17)	**South Africa** (n=14)	**Tanzania** (n=16)
				**Action (for E-MOTIVE team):** proposed is to use stickers, to be designed and developed
**PPH TROLLEY**				
**Concept of Trolley with PPH kit**<br><br>**Q1. To what extent might assembling all of the E-MOTIVE supplies in one place help to deliver the bundle quickly?**	Have trolley in Makueni and checklist of content	Assembling the supplies would help to deliver the bundle quickly	Already in place across sites	Having the supplies all in one place is going to be useful
**Stocking of Trolley**<br><br>*Q2. How can we ensure the trolley is consistently stocked?*<br><br>*(Options given)*	Mixed views<br><br>**Action (for E-MOTIVE Team)**: contact sites using Trolley/Kit for input	Mixed views<br><br>**Decision**: Every time an item is used it is restocked	Mixed views<br><br>**Decision**: Combination of one person assigned and every time an item is used it is restocked<br><br>**Follow-up working group meeting**:<br><br>It is standardized across sites that the PPH box is checked daily and restocked after each use, in one AC site they tape the box closed (easy to open) as a way of confirming the PPH box is stocked and ready for use.	Mixed views<br><br>**Decision**: use existing checking procedures
**Refrigeration of Oxytocin**<br><br>*Q3. What can we do about oxytocin, which needs refrigeration?*	Keep in fridge<br><br>Place empty bottle of oxytocin in Trolley/Kit to act as a reminder	Protocol requires oxytocin to be kept refrigerated.<br><br>**Action (for E-MOTIVE team)**: find a locally appropriate solution (cool box or fridge) and temporary solutions from power cuts	All labour wards have fridges and oxytocin should be kept in the fridge where people know to find it	Keep in fridge and take out a small supply from fridge in vaccine carrier with ice packs. Use ice packs for sites with no refrigeration in labour ward

**Question**	**Kenya** (n=16)	**Nigeria** (n=17)	**South Africa** (n=14)	**Tanzania** (n=16)
**Essential supplies & equipment**<br><br>*Q4. Should we limit the trolley kit to the essential supplies and equipment to deliver the E-MOTIVE intervention, or include other items?*<br><br>*(Options given)*	Mixed views<br><br>**Action (for E-MOTIVE Team**): modular trolley design which can be adapted to the needs of each setting	A PPH Trolley Kit which includes all the things needed for the E-MOTIVE bundle and refractory PPH (e.g., tamponade device, speculum, instruments)	Mixed views<br><br>**Follow-up working group meeting** PPH box to accompany the existing emergency trolley which has instruments etc. Sites that already use a PPH box, they currently already have most items required for MOTIVE except TXA. The current PPH boxes will be adapted to include TXA. New PPH box will be introduced to accompany the emergency trolley at facilities that do not have one already	An E-MOTIVE PPH Bundle trolley kit-holding only the items to deliver the E-MOTIVE bundle
**Portable vs Stationary Trolley**<br><br>*Q5. Should the E-MOTIVE Emergency PPH kit be stationary or portable?*	Accommodate both trolley & kit option to make it fit for purpose for the setting	A movable trolley	Portable carry case/box	Portal trolley but different preferences based on size of wards. For smaller wards with limited space prefer carry case
**Checklist**<br><br>**Q6. Is there an existing checklist currently used for checking off stock?**	**Action (for E-MOTIVE Team):** obtain existing checklists from the sites to collate ideas and to produce a checklist	There is an existing checklist system in place (E-MOTIVE team to review existing checklist to adapt if required)	There are checklists for PPH boxes and emergency trolleys<br><br>Should be checked at the beginning of every shift, using a checklist tool	Some have checklists and some do not. For those who do, the current checklist can be adapted
**TRAINING**				
**Frequency of training**<br><br>**Q1. How can training be organised to avoid disrupting routine care?**<br><br>**(Options given)**	Training in week one, and re-training every month	Agreement on all staff to be trained within one week (or the shortest possible time frame on consecutive days), then every 2 months (to fit in with the timeline of E-MOTIVE study).	No consensus - once a month is unlikely to be feasible	No consensus on the frequency of training (every two-week or once a month).<br><br>Needs to consider the number of staff (as shortage of staff and it may hinder work at the facility if most/all staff attend training).

**Question**	**Kenya** (n=16)	**Nigeria** (n=17)	**South Africa** (n=14)	**Tanzania** (n=16)
				**Solution**: Proposed training should be provided in groups - if one group attends the training, the remaining group should continue work.
**Q2. Frequency of practices**<br><br>**How often do you think your staff can do these short activities after training?**<br><br>**(Options given)**	Once a week after initial training	It will have to fit in and be adapted to different sites, i.e., frequency variation across facilities, and this might also change over time.	Once per week	Once a week
**Space for practices**<br><br>**Q3. What is the best way to support the practice activities?**	Preference for separate room	The best option could be to have the simulator in the ward so that staff can go in pairs to practice at appropriate times during their shift.	**Follow-up working group meeting:**<br><br>What works best for the healthcare facility. Needs to be a site-specific decision	No discussion
**Training of new staff**<br><br>**Q4. How can new staff best be trained when they join the ward?**	Have one on one training include in practice sessions	Immediate training for new staff by existing staff; turnover is high (i.e., new doctors every month but they should have been trained beforehand) & nurses trained before starting in ward.	Mixed views<br><br>**No decision reached**	Have one on one training. Local champion and the facility can train the new staff
**Use of TXA by nurses**<br><br>**Q5. What barriers do you think providers will face in using TXA and how do you think we can best overcome them?**<br><br>**Can everyone attending birth give TXA?**<br><br>**Does anyone have experience with TXA?**	In protocols/guidelines that nurses can administer TXA	In tertiary facilities only doctors give TXA, in secondary facilities doctors and nurses manage as a team.<br><br>Training is needed as doctors give IV injection and nurses and midwives are not trained for this intervention.	Nurse/midwives can give TXA if there is a standing order prescription, a protocol may be required	If the midwife or nurse has training on administering TXA can implement it without doctor's prescription.

Supplement to: Forbes G, Akter S, Miller S, et al. Development and piloting of implementation strategies to support delivery of a clinical intervention for postpartum hemorrhage in four sub-Saharan Africa countries. *Glob Health Sci Pract*. 2024;12(5). https://doi.org/10.9745/GHSP-D-23-00387

Question	**Kenya** (n=16)	**Nigeria** (n=17)	**South Africa** (n=14)	**Tanzania** (n=16)
**CHAMPION**				
**Attributes of a local champion**<br><br>**Q1. What attributes and abilities will you look for in a champion?**	Knowledgeable, have good interpersonal skills, passionate and interacts well with all cadres of staff (clinical & managerial)	Should be an influencer; knowledgeable about the burden of PPH and other related aspects; capable of doing things that will be effective and can enforce things, must have a strategic position; passionate, disciplined, diligent and well-motivated; needs to have commitment to patient care in addition to clinical knowledge of the PPH bundle management	Q not asked because of time	Hard-working, a team player, good communication and leadership skills, knowledgeable about PPH, able to resolve conflicts and to convince use of drape & bundle
**Effective working**<br><br>**Q2. What could facilitate or hinder the effective working of a champion?**<br><br>**How can we tailor the roles of local champions to make it most effective for E-MOTIVE implementation?**	Barriers: administrators/managers not understanding their role<br><br>Enablers: favourable working relationship at District level and within the team	Provide guidance for local champion	Hindrances are understaffing, coping with responsibility and not being paid extra (W Cape) and not receiving specific training (WC).<br><br>KZN against extra payment	Hindrance is not being knowledge about PPH
**Number and cadre of local champion**	Two:<br><br>a midwife/nurse and a doctor	Two:<br><br>a midwife/nurse and a doctor	Two:<br><br>a midwife/nurse and a doctor	Two:<br><br>a midwife/nurse and a doctor
**AUDIT AND FEEDBACK (A/F)**				
**Feasibility of A&F**<br><br>**Q1. To what extent is audit and feedback on PPH outcomes likely to be feasible in the study sites?**	Not a huddle/debrief format; give feedback at existing meetings	Good idea will benefit practice. Over the years of receiving feedback already has improved skills, people take ownership and see benefits	A & F is feasible	Feasible and will be helpful. Some facilities are planning to initiate weekly meeting that are similar to A&F.

**Question**	**Kenya** (n=16)	**Nigeria** (n=17)	**South Africa** (n=14)	**Tanzania** (n=16)
**What to audit & give feedback on**<br><br>**Q2. What information on current PPH practice would be most useful and motivating for staff to know?**<br><br>**(Options given)**	Data on outcomes: Vaginal birth rates; C-section rates; # Postpartum maternal deaths; # Postpartum laparotomies; # Postpartum blood transfusions; # Severe PPHs / Severe PPH rate and PPH detection rate<br>Data on compliance with bundle: Overall adherence to E-MOTIVE (overall % compliance with all components of bundle for cases of PPH detected and overall % compliance broken down by component of E-MOTIVE bundle	Data on outcomes: Vaginal birth rates; C-section rates; # Postpartum maternal deaths; # Postpartum laparotomies; # Postpartum blood transfusions; # Severe PPHs / Severe PPH rate and PPH detection rate<br>Data on compliance with bundle: Overall adherence to E-MOTIVE (overall % compliance with all components of bundle for cases of PPH detected and overall % compliance broken down by component of E-MOTIVE bundle **plus** additional ones e.g., assess outcome of intervention	Data on outcomes: Vaginal birth rates; C-section rates; # Postpartum maternal deaths; # Postpartum laparotomies; # Postpartum blood transfusions; # Severe PPHs / Severe PPH rate and PPH detection rate<br>Data on compliance with bundle: Overall adherence to E-MOTIVE (overall % compliance with all components of bundle for cases of PPH detected and overall % compliance broken down by component of E-MOTIVE bundle **plus** near misses and referrals into the site.	Data on outcomes: Vaginal birth rates; C-section rates; # Postpartum maternal deaths; # Postpartum laparotomies; # Postpartum blood transfusions; # Severe PPHs / Severe PPH rate and PPH detection rate<br>Data on compliance with bundle: Overall adherence to E-MOTIVE (overall % compliance with all components of bundle for cases of PPH detected and overall % compliance broken down by component of E-MOTIVE bundle
**Preference for level of performance to measure**<br><br>**(Options given)**<br><br>**Q3. At what level should performance be measured and fed back?**	Mixed views<br><br>**Decision**: All E-MOTIVE facilities and facilities, but not at team level	Mixed views<br><br>**Decision**: a combination of national level data (all Nigerian facilities) and facility level data	Mixed views<br><br>**Follow-up working group meeting**: facility level	**Decision**: At the facility level of performance because this will include team performance.
**Recipients of feedback**<br><br>**Q4. Which staff should receive this feedback?**<br><br>**(Options given)**	Everyone including managerial/admin staff	All people involved in the study, all roles including admin should receive this feedback monthly; All staff involved in managing to assess effectiveness and share with admin beyond site	Q not asked	Everyone including managerial/admin staff to improve the outcomes
**Format of feedback**<br>**Q5. How should we deliver this feedback?**<br><br>**(Options given)**	A combination of visually (in graphs and charts), written summaries and verbally during meetings<br><br>**Plus** displayed in the wards	A combination of visually (graphs, charts) and written summaries, i.e., have hard copies to be used by local champion	Mixed views<br><br>**Decision**: A combination of visually (in graphs and charts), written	A combination of visually (in graphs and charts), written summaries and verbally during meetings plus displayed in the wards

Supplement to: Forbes G, Akter S, Miller S, et al. Development and piloting of implementation strategies to support delivery of a clinical intervention for postpartum hemorrhage in four sub-Saharan Africa countries. *Glob Health Sci Pract*. 2024;12(5). https://doi.org/10.9745/GHSP-D-23-00387

**Question**	**Kenya** (n=16)	**Nigeria** (n=17)	**South Africa** (n=14)	**Tanzania** (n=16)
	Consider WhatsApp group		summaries and verbally during meetings (n=6/9)	**Plus** consider using the dashboard to track adherence and feedback may be through meetings
**Frequency of feedback**<br><br>**Q6. How often should feedback be provided to facilities?**<br><br>**(Options given)**	**Decision**: Monthly	**Decision**: Monthly to fit in with monthly team meetings	Mixed views<br><br>**Decision**: every 6 months (n=4/7)	**Decision**: Monthly basis meeting (given sufficient data)
**Current practice Comparisons Q7. What should current practice be compared to, in order to most motivate staff to improve practice?**<br><br>**(Options given)**	A benchmark or standard of care (e.g., 90% adherence to E-MOTIVE intervention) and other facilities of a similar size and case mix plus changes over time	A benchmark or standard of care (e.g., 90% adherence to E-MOTIVE intervention) and change over time within the facility	Mixed views<br><br>**Decision**: A benchmark or standard of care (e.g., 90% adherence to E-MOTIVE intervention)<br><br>(n=10/18)	Other facilities in the same region
**Use of feedback**<br><br>**Q8. What do you think facilities would do with this feedback?**	Use this information to improve the quality of their performance	Improves services, identify right and wrong, tell admin where improvements are needed, success or failure of changing practice	Use at meeting to demonstrate what is working well and for team to agree on require changes	Use to improve personal skills

Supplement to: Forbes G, Akter S, Miller S, et al. Development and piloting of implementation strategies to support delivery of a clinical intervention for postpartum hemorrhage in four sub-Saharan Africa countries. *Glob Health Sci Pract*. 2024;12(5). https://doi.org/10.9745/GHSP-D-23-00387

SUPPLEMENT 3. Mapping of implementation influences to TDF domains including supporting quotes (Enablers in green; barriers in red and a combination of enabler and barrier in yellow)

E-MOTIVE intervention and implementation strategies	Implementation influences grouped by TDF domain	Kenya n=15	Nigeria n=15	South Africa n=14	Tanzania n=14	Supporting Quotes
Calibrated drape	**Environmental context and resources**					
	All sites have an adequate supply of calibrated drapes over all shifts including weekends					*No, all the days are the same. (laugh) The days of the week does not have an influence (Midwife, South Africa)*
	Use of the drape can be negatively affected by a lack of appropriate beds having a long term, post-trial supply of the drape by the facility and the disposal of used drapes					*Sometimes the only thing we do encounter sometimes is that for instance in the lying in room, the bed is just straight not like the one in second stage room that you can remove the lower part of it so that the drape will go down (Midwife, Nigeria)*
	It can be more challenging to place the drape under the woman when she is unconscious, overweight or wants to give birth on the floor					*The difficulty depends on the patient's condition, the patients' peculiarities…. If the patient is morbidly obese (Doctor, Nigeria)*
	Use of the drape can be negatively affected by a lack of appropriate beds having a long term, post-trial supply of the drape by the facility and the disposal of used drapes					*All the three beds are full (Midwife, Tanzania)*
	**Beliefs about consequences**					
	Beliefs that PPH detection is earlier and quicker using the calibrated drape and despite initial scepticism, staff acknowledge that the drape can improve accuracy of estimated blood					*The drape it will show you the amount and will help to detect PPH early and start the management as the way EMOTIVE bundle tells. Therefore, it has improved us (Midwife, Tanzania)*
	Beliefs that using the calibrated drape results in an accurate measurement of blood loss because there is no more visual estimation of blood loss, and uncertainty about the volume of blood loss as to whether or not it is a PPH					*Having the calibrated drape makes PPH detection easier because the volume of blood loss is visual (Midwife, Kenya)*

Supplement to: Forbes G, Akter S, Miller S, et al. Development and piloting of implementation strategies to support delivery of a clinical intervention for postpartum hemorrhage in four sub-Saharan Africa countries. *Glob Health Sci Pract*. 2024;12(5). https://doi.org/10.9745/GHSP-D-23-00387

**E-MOTIVE intervention and implementation strategies**	**Implementation influences grouped by TDF domain**	**Kenya n=15**	**Nigeria n=15**	**South Africa n=14**	**Tanzania n=14**	**Supporting Quotes**
	Beliefs that the number of PPH cases has increased since using the calibrated drape was introduced					*The frequency of detection has increased (Clinical Admin, Nigeria)*
	**Social influences**					
	Keeping the drape in place depends on the woman accepting that a drape is required to measure blood loss and some women do not accept drape because it is made of plastic therefore uncomfortable					*Enabler: I cannot pinpoint anything it is only that they know that it is for their own good (Midwife, Nigeria)*<br><br>*Barrier: They do not like it…. It's wet…. And it is cold also, the patients have told me the plastic is cold underneath them (Midwife, South Africa)*
	Accuracy of blood collection can be affected by overweight women and women moving about, i.e., not maintaining a stable position					*For the women maybe let me say the overweight… women. sometimes when you put the bundle…. when the blood is flowing because you can't tie sometimes it's difficult to tie the woman maybe on the up to the abdomen. So as maybe they are trying to move around because during labour some women try to move, they cannot be stable. So sometimes the blood can spill out (Midwife, Kenya)*
	Research staff can take an active part in placing of the calibrated drape					*Like the research midwife sometimes feels it is a personal thing…. she feels it seems that she must be the one to do it. so, it got to a stage that most of us just fold our arms and watch her (Nigeria, Midwife)*
	**Behaviour regulation**					
	Having the calibrated drape makes PPH detection easier because of the measurement line and the volume of blood loss is visual					*Use of drape from that line you already know that the patient is bleeding….it is also easier because when you can also use it instead of visual estimation (Midwife, Nigeria)*
	Detecting a PPH by monitoring the vital signs is easier and quicker using the cradle device					*This one [cradle device] makes that at least you can save time and easy to help them out. (Midwife, Kenya)*
	**Skills**					
	Accuracy of blood collection can be affected by the drape not being tied; accuracy of recognizing blood loss volume could be affected by HCPs not					*The blood is flowing because you can't tie sometimes it's difficult to tie the woman maybe on the up to the abdomen… maybe they are trying to*

Supplement to: Forbes G, Akter S, Miller S, et al. Development and piloting of implementation strategies to support delivery of a clinical intervention for postpartum hemorrhage in four sub-Saharan Africa countries. *Glob Health Sci Pract*. 2024;12(5). https://doi.org/10.9745/GHSP-D-23-00387

E-MOTIVE intervention and implementation strategies	Implementation influences grouped by TDF domain	Kenya n=15	Nigeria n=15	South Africa n=14	Tanzania n=14	Supporting Quotes
	elevating the funnel to read the calibrations when woman lying flat on					*move around because during labour some women try to move, they cannot be stable (Midwife, Kenya)*
	**Beliefs about capabilities**					
	Beliefs that it can be more difficult to correctly hang the drape when it is a smaller bed					*the couch [bed] which cannot stable with…. maybe they are too small. …... the woman cannot to extend…. because they are those coaches [beds] which you can have the drape hanging down (Midwife, Kenya)*
	It can be more challenging to use the drape and monitor vital signs when working alone					*Enabler: We use drapes for our patients rigorously and they are calibrated... we do vital signs (Midwife, Nigeria)*<br>*Barrier: Most times I don't check vital signs when I am alone, because I used to feel going to waste time checking vital signs the woman will still be bleeding more (Midwife, Nigeria)*
	**Knowledge**					
	Beliefs that measuring blood loss using the drape alone is not the only indicator of a PPH; other indicators are vital signs and the woman's condition					*We usually depend on clinical signs and symptoms which doesn't actually give us a sense of direction because of patient peculiarities but the calibrated drape gives us an idea, it kind of gives us a general overview and it actually helps us in our way of detection PPH (Doctor, Nigeria)*
	Some staff are confused about whether to place the drape on top of a linen or to remove the linen					*The thing is, also, we must also think of what is right? Would you really now put that plastic on top of the soiled linen? Or what is the right thing to do? Isn't it not the right thing to remove the source linen? (Midwife, South Africa)*
	**Social/Professional Role & Identity**					
	Having the drape provides the evidence to support a midwife's diagnosis versus a higher-level professional guesstimate					*We are trying to tell them this patient is bleeding but they [Doctors] are not seeing it from our own angle. But now with the use of drapes there is nothing to hide. (Midwife, Nigeria)*
	**Social influence**					

E-MOTIVE intervention and implementation strategies	Implementation influences grouped by TDF domain	Kenya n=15	Nigeria n=15	South Africa n=14	Tanzania n=14	Supporting Quotes
MOTIVE components	Managers are supportive by ensuring that the necessary drugs are available, and the trolley is re-stocked and demonstrating how to use the bundle					*If you tell the administration that we need a certain drug they help……. So, they give us support (Midwife, Tanzania)*
	On the whole colleagues are supportive by coming to assist when called; however, some colleagues can be less supportive					*Supportive in terms of when you call for help, anybody there is on duty, anybody that is on duty post and call for help, the response is there. (Doctor, Nigeria)*
	Some health care providers stated a barrier to implementation existed if the woman is 'upset or in trauma' from PPH, does not want to be examined, does not want to have a cannula inserted, are 'un-cooperative' about massage because it is 'painful', or the woman has mental health issues or has been referred in a 'worst state' or traditional beliefs					*We face challenges, when handling Maasai and Mang'ati; sometimes they have tear but they refuse to be repaired, sometimes we have to consult their relative (Midwife, Tanzania)*
	Team-working is necessary to effectively use the bundle; delivering each component needs to be delegated (usually by the primary provider) or decided as a team so it is challenging to implement MOTIVE if you are working alone					*If an individual is alone, you can deliver it alone... but like here where I am, there is always a doctor on call, there is first on call, second on call, they're always there... And midwives like here, we're always two; it's in exceptional cases that it is one person that would be there (Midwife, Nigeria)*
	Communication has improved because team-working is essential to implement the bundle					*It has affected us positively because we are able to communicate effectively in management of PPH… everyone knows whatever Emotive stands for and having a team leader that tells you to do something. You're not left hanging up at like you do not know what to do. So, communication is very effective right now (Admin, Kenya)*<br><br>*Communication is not. It is not different as we used to (Midwife, South Africa)*

Supplement to: Forbes G, Akter S, Miller S, et al. Development and piloting of implementation strategies to support delivery of a clinical intervention for postpartum hemorrhage in four sub-Saharan Africa countries. *Glob Health Sci Pract*. 2024;12(5). https://doi.org/10.9745/GHSP-D-23-00387

E-MOTIVE intervention and implementation strategies	Implementation influences grouped by TDF domain	Kenya n=15	Nigeria n=15	South Africa n=14	Tanzania n=14	Supporting Quotes
	Replacing plastic drape with another material because woman dislike them); having more weekly huddles					*We probably could discuss it on the huddles every week. And that's possibly one thing to make people more aware (Doctor, South Africa)*
	Belief that informed consent from the woman is required in advance before delivering the bundle; after counselling woman can be persuaded					*Most times we don't seek their consent, we tell them it is part of their treatment. We don't counsel them separately on EMOTIVE, we just add it to normal delivery care (Midwife, Nigeria)*
	**Emotion**					
	Using the intervention has been effective at reducing stress and panic for HCPs because of more positive outcomes					*This helps in simplifying the management as you can even handle it alone without panicking because the mother is not in shock. You just relax and save the mother (Midwife, Tanzania)*
	**Beliefs about capabilities**					
	Self-confidence in managing a PPH or it has increased since the introduction of the EMOTIVE intervention; equally confident in all delivering all components					*Emotive has brought some confidence ... for me (Midwife, Kenya)*
	It is easy to implement all components in the bundle					*I see that when you use this bundles, it's better and easier (Doctor, Tanzania)*
	**Beliefs about consequences**					
	The intervention has been effective at improving and increasing the PPH detection and management and at reducing maternal mortalities					*It improves the management, it improves dramatically. It's been improved (Midwife, South Africa)*
	Different views on how much using the bundle has changed the management of PPH					*I guess with any change... then any new introduction, if people would tend to be a bit sceptic, but they have come to accept and actually use the bundle and also embrace the use of the bundle as a way of managing PPH (Midwife, Kenya)*

E-MOTIVE intervention and implementation strategies	Implementation influences grouped by TDF domain	Kenya n=15	Nigeria n=15	South Africa n=14	Tanzania n=14	Supporting Quotes
	**Social/Professional Role & Identity**					
	It is within the scope (role) of midwives to deliver all MOTIVE components, unless it is cervical tear causing the PPH then a doctor would be called to do the examination					*Enabler: Emotive give us [midwives] the permission or authority to go ahead with the bundle as long as we have detected a PPH (Midwife, Nigeria)*<br><br>*Barrier: We examined, so based on our examination then we're able to suture the tear and we're able to control the postpartum haemorrhage (Doctor, Nigeria)*
	Use of tranexamic acid is the least liked component of the bundle, possibly because many healthcare providers had never used it before or had some hesitancy to use without advice or being prescribed by doctors					*They support us firstly they did the protocol for giving tranexamic acid as midwives. So, it helps now, not to wait for the doctor to come in ward.* (Midwife, Nigeria)
	**Memory, attention and decision-process**					
	Some healthcare providers can forget to implement the whole intervention (including the drape) whilst others always remember; helpful reminders are the EMOTIVE acronym and having charts and poster on display					*Yes, there are wall charts/ diagrams of EMOTIVE pasted around the bedside, once you enter our labour ward that is one of the conspicuous charts you see. Once you enter the labour ward you don't need to keep remembering (Doctor, Nigeria)*<br><br>*Sometimes staff use the drape but forget to measure the blood loss (Doctor, Nigeria)*
	**Environmental Context & Resources**					
	Implementing all components of the bundle requires manpower, mostly it is 2 people on a shift. At times when it is busy, working alone happens, i.e., have no assistance					*At night… over weekends when we don't have a full complement of staff. If the labour board becomes very busy, then it can be difficult to give the correct amount of attention to every patient (Doctor, South Africa)*
	Require plentiful supply of drapes and the layout of the labour/delivery rooms; continued supply of all components and trolley particularly the cost, modification of beds to properly use the					*I think in our hospital for it to be to be sustainable and continuous. I think the liability of the threats but so far, they haven't been available all along (Doctor, Kenya)*

E-MOTIVE intervention and implementation strategies	Implementation influences grouped by TDF domain	Kenya n=15	Nigeria n=15	South Africa n=14	Tanzania n=14	Supporting Quotes
	drape and expand to the lower-level facilities which refer to this facility					
	Despite initial reluctance to implement the intervention, staff are using it more including at night which was more problematic in the beginning because of a lack of supervision					*I will say even people gradually come to accept and understand the Emotive bundle...now people have accepted the use of the drape and they're using it quite often almost for every delivery (Midwife, Kenya)*
	Implementing the bundle is timesaving for the healthcare providers					*We would do maybe one thing at a time and we wait for it to act and then continue with the next one, for example, ...massaging the uterus and then the blood... if the bleeding stop, you stop everything. And then you find that maybe after five minutes, the woman will bleed again that is when we would give oxytocin. So, it has ... it takes less time right now (Clinical Admin, Kenya)*
	Tranexamic acid has been omitted when it is out of stock at the pharmacy					*We have. We ran out of [tranexamic acid] (Midwife, Kenya)*
	Some healthcare providers felt using the bundle took more time and energy involving the whole team whilst others felt less effort was needed because everything (i.e., drugs and consumables) were readily available					*Enabler: It doesn't require any additional effort (Doctor, Nigeria)*<br><br>*Barrier: I think the emotive bundle take more time and energy (Midwife, Nigeria)*
	**Skills**					
	Informing women about the drape [consent obtained] earlier					*We explained first before putting to them and we seek content from them (Midwife, Kenya)*
	Training of new house officers and registrars					*Well, we have been trained, the new house officers, the new registrars maybe need more training (Doctor, Nigeria)*
	All healthcare providers have expertise in delivering all components of the bundle regardless of role as a midwife or doctor					*So, it's that team…. will help both [midwife and doctor] they will…. understands it's very clear (Midwife, Kenya)*
	**Knowledge**					

E-MOTIVE intervention and implementation strategies	Implementation influences grouped by TDF domain	Kenya n=15	Nigeria n=15	South Africa n=14	Tanzania n=14	Supporting Quotes
	[Not] learning from the experiences of other facilities about implementing the whole intervention					*My contribution is for them to keep expand it, sustain it and probably also use it in secondary health facilities because most of the patients we see here, are actually referred from primary and secondary health centres (Doctor, Nigeria)*
	**Goals**					
	It is important to use all component of the bundle					*Give the bundle within 15 minutes, so all components can be given simultaneously (Midwife, Tanzania)*
	Healthcare providers have a target of aiming to implement the bundle for all cases of PPH					*.... when there is a chance or there is availability of Emotive equipment, I would rather keep on using the Emotive (Midwife, South Africa)*
	**Intentions**					
	Some healthcare providers are reluctant about using the bundle so require more encouragement to increase its uptake					*I will say even people gradually come to accept and understand the Emotive bundle...now people have accepted the use of the drape and they're using it quite often almost for every delivery (Midwife, Kenya)*
Training and practice sessions	**Skills**					
	Training was educational and interactive; the scenarios were adequate to learn about using EMOTIVE effectively					*The scenario helps us to practice as if we are in real situation. So, it made us... it gave us an opportunity to practice with the mannequin so that we can be able to deliver exactly what is needed when the patient needs our attention (Clinical admin, South Africa)*
	Recommend having more training and skills sessions or would like to have less training and skills sessions					*Enabler: We still need to elaborate more…although we are being trained on how to use the tranexamic acid because before we do not give the tranexamic acid, giving the Misoprostol and all of that... It seems there are still new things that we still need to know (Midwife, Nigeria)*<br><br>*Barrier: We don't need to practice, every day we have PPH that is our practice system (Midwife, South Africa)*
	Beliefs that practice sessions of real cases are beneficial for improving PPH management or					*Enabler: Personally felt that received sufficient bundle training (Midwife, Tanzania)*

E-MOTIVE intervention and implementation strategies	Implementation influences grouped by TDF domain	Kenya n=15	Nigeria n=15	South Africa n=14	Tanzania n=14	Supporting Quotes
	sufficient practice comes from managing real PPH cases in the labour ward					*Barrier: Not really practice, but we've had real cases on the ground. We believe that that was enough (Doctor, Kenya)*
	Beliefs that the training has improved the skills needed to detect and to manage a PPH					*It has also improved my skills. Previously, I wouldn't say that I could handle a PPH but now I can (Midwife, Kenya)*
	Beliefs that team-working including communication has improved after training because each person knows how we can manage as a person within in a team					*I think it [teamwork and communication] has improved because what usually happen before is that we are trying to tell them this patient is bleeding but they are not seeing it from our own angle. But now with the use of drapes there is nothing to hide (Midwife, Nigeria)*
	Beliefs that the training was effective at changing practice e.g., no longer detecting using visual estimation, asking mother to do the massage					*It feels very good. Because you're able to know where you are at the point ... you're able ...you are encouraged on what you've been doing. And if any changes that you need to make.... Because you're able to change where you need to (Midwife, Kenya)*
	**Beliefs about capabilities**					
	Beliefs that the skills sessions are effective at building confidence in using the intervention quickly and efficiently					*They have increased my ability in early detection of PPH, also has influenced my confidence in providing services (Midwife, Tanzania)*
	**Memory, attention and decision-process**					
	Beliefs that the initial training acted as a refresher for the detection and management of PPH and practice sessions were effective reminders of what may have been forgotten after the initial training					*Practical training is very good, because it gives you a clear picture of how to provide services (Midwife, Kenya)*
	**Environmental context and resources**					
	Providing training had an influence on the system as it was challenging to incorporate training for new staff					*Doing the drills because not all the time or something. The other midwife is busy, maybe later. You have change there as the student there (Midwife, Kenya)*
PPH	**Environmental context and resources**					

Supplement to: Forbes G, Akter S, Miller S, et al. Development and piloting of implementation strategies to support delivery of a clinical intervention for postpartum hemorrhage in four sub-Saharan Africa countries. *Glob Health Sci Pract*. 2024;12(5). https://doi.org/10.9745/GHSP-D-23-00387

E-MOTIVE intervention and implementation strategies	Implementation influences grouped by TDF domain	Kenya n=15	Nigeria n=15	South Africa n=14	Tanzania n=14	Supporting Quotes
Trolley	Beliefs that responding to a PPH is quicker when all essential drugs, equipment and consumables are in the one place (i.e., fewer delays)					*Since everything is one place, it saves a lot of time (Midwife, Kenya)*
	Belief that having E-MOTIVE trolley fully stocked will encourage people to use it					*it is helpful, because if we could have left them out, you might find that they are not there, but with the use of the trolley we have a checklist which quickly shows s available drugs. It has all medications, but sometimes tranexamic acid is not there (Midwife, Tanzania)*
	Belief that it is easy to access the trolley/carry case unless there is a lack of space in the delivery room/labour ward					*We usually have a PPH kit……. you carry and put it in a trolley…it is easy for you to administer [than a trolley] (Midwife, Nigeria)*
	Reasons given for not using the E-MOTIVE trolley were: (1) being busy & not having enough staff (competing tasks); (2) and the drugs are not kept in the trolley					*I will waste my time bringing it out, pulling it and struggling to push it back. That's why I don't even go near the trolley but when you bring something nice, perfectly working in good shape, that is good and not as wide as the one we're having now, maybe it will help (Midwife, Nigeria)*
	Reasons given for not using the E-MOTIVE trolley were (1) considered a waste of staff's time and (2) not in good working order					*Most times when I bring this one, pushing it back is a problem. I will waste my time bringing it out, pulling it and struggling to push it back. That's why I don't even go near the trolley but when you bring something nice, perfectly working in good shape, that is good and not as wide as the one we're having now, maybe it will help (Midwife, Nigeria)*
	Shortages of supplies because of time delays (e.g., 24 hours) after placing order request					*But at times, we get out of stock and we have to wait for this supply to be made. We request and they will now give us (Midwife, Nigeria)*
	**Beliefs about consequences**					
	Having the E-MOTIVE trolley makes managing a PPH easier and it can require less effort from the healthcare provider					*It's easy because you don't have to run around looking for stuff that you need to use and, in the box, you get everything (Clinical Admin, South Africa)*
	Beliefs that having the E-MOTIVE PPH box is important because it can improve PPH management					*It improved because the fast you act the best you get your result (Midwife, Nigeria)*

Supplement to: Forbes G, Akter S, Miller S, et al. Development and piloting of implementation strategies to support delivery of a clinical intervention for postpartum hemorrhage in four sub-Saharan Africa countries. *Glob Health Sci Pract*. 2024;12(5). https://doi.org/10.9745/GHSP-D-23-00387

**E-MOTIVE intervention and implementation strategies**	**Implementation influences grouped by TDF domain**	**Kenya n=15**	**Nigeria n=15**	**South Africa n=14**	**Tanzania n=14**	**Supporting Quotes**
	**Knowledge**					
	Staff (including non-clinical staff) were aware about the E-MOTIVE trolley, which meant that the appropriate drugs/supplies were provided when called for by the health worker	[green]				*I can say even someone who's probably cleaning around you can be able to tell them 'Can you please hand me that kit?' because you know everything is on that kit as compared to telling someone 'give me oxytocin?' and probably they are not aware what oxytocin is (Midwife, Kenya)*
	Limited knowledge about PPH trolley (discussing instead a combined emergency & PPH tray)		[red]			*I don't know; we have our emergency tray, well, where we got drugs including TXA, we have the oxytocin we keep in the fridge (Midwife, Nigeria)*
	**Memory attention and decision-process**					
	Frequent use of E-MOTIVE trolley was reported, whilst acknowledging not used all the time	[amber]	[amber]	[amber]	[amber]	*Most of the time it has been rendered useless because the medication to be kept there are not available*<br>*it is not mostly used because sometimes it lacks some things (Doctor, Tanzania)*
	**Intentions**					
	Reasons given for not using the E-MOTIVE trolley were individuals not wanting to use it	[red]				*We don't always make use of that trolley, most times. Truth be told we don't use it (Midwife, Kenya)*
Local champion	**Social/Professional role and identity**					
	The support offered by the champion includes ensuring there is sufficient stock of the bundle components including the drape for night shifts, training new staff, encouraging use of the bundle, helping staff to use the whole intervention and frequent communication to ensure a coordinated team	[green]	[green]	[green]	[green]	*They are able to train them [the staff] and any questions that are there, they are able to answer. ..... having a champion will help them get frequent updates of the same. Of the bundles that is management of PPH. (Midwife, Kenya)*<br><br>*Also, in the supervision of an emergency trolley so that the trolley should be having all the materials (Midwife, Nigeria)*
	**Beliefs about consequences**					

E-MOTIVE intervention and implementation strategies	Implementation influences grouped by TDF domain	Kenya n=15	Nigeria n=15	South Africa n=14	Tanzania n=14	Supporting Quotes
	Having a champion brings benefits, therefore would recommend having a champion at other facilities to improve PPH management					*There must be the person that would be overseeing, monitoring and supervising and encouraging... it is very important (Doctor, Nigeria)*
	**Social Influence**					
	It is important to have a 'focal' person to go to if you need clarification or can talk to the staff about remembering to use the intervention and that they are a member of the clinical team in the dept					*Because the person who's supporting will come and check us when doing the management of PPH and if we are doing mistakes, she will be definite for a certain day. Another thing she will even praise us if we did manage it well (Midwife, South Africa)*
	Initial misunderstandings by some staff that the champion might be receiving incentives and not sharing it with others in the team/shift					*Grudges came from feeling that some have been paid to do this job, when they're always not on ground to do it while it is others are doing the job when they take the credit. Such things came up (Midwife, Nigeria)*
	**Environmental context and resources**					
	Concerns about having a champion include having the capacity to carry out the role due to competing clinical tasks, equal division of responsibilities between 2 champions, and working on a different shift from other staff so not available to everyone					*in order to achieve this very well, maybe you need someone, you need to get someone who will be on ground just to do that work, not that the person will combine two (Midwife, Nigeria)*
Audit and feedback	**Beliefs about consequences**					
	Belief that receiving feedback on clinical practice is useful because it shows the performance on the bundle and where improvements are needed; it can be motivating					*Motivates us [receiving feedback] to do better (Midwife, Kenya)*
	**Goals**					
	Beliefs that audits are important for setting targets and the dashboard for showing progress					*I think this has been helpful because you have been able to set target; we've been able to see our progress...see how many cases we've had... And how many of those have you been able to achieve and whether it*

Supplement to: Forbes G, Akter S, Miller S, et al. Development and piloting of implementation strategies to support delivery of a clinical intervention for postpartum hemorrhage in four sub-Saharan Africa countries. *Glob Health Sci Pract*. 2024;12(5). https://doi.org/10.9745/GHSP-D-23-00387

E-MOTIVE intervention and implementation strategies	Implementation influences grouped by TDF domain	Kenya n=15	Nigeria n=15	South Africa n=14	Tanzania n=14	Supporting Quotes
						has been an improvement compared to the previous months (Doctor, Kenya)
	**Environmental context and resources**					
	Tranexamic acid is locked away					*Sometimes during weekend or during shifts, sometimes you the drugs or the tranexamic acid might not be accessible, because it's locked in the store (Midwife, Nigeria)*
	Belief that audit and feedback is important, therefore should be introduced at other facilities					*Get knowledge and impact to one another, and seek wisdom in managing that decision (Midwife, Kenya)*
	All staff should receive audit and feedback by putting up posters and setting up a WhatsApp group					*Maybe you can put the poster up…..if you can put up so that everybody can see (Midwife, South Africa).*
	Feedback is helpful because it tells you if there is any 'stock out					*It allows me to know the amount of medical supplied used to help in delivery such as oxytocin, tranexamic acid, and we can identify if there is any stock out (Doctor, Tanzania)*
	**Skills**					
	Belief that differences in use of oxytocin and tranexamic acid displayed in the dashboard compared to actual use is because of poor documentation					*Maybe issues of documentation (Midwife, Kenya)*
	The feedback can inform practice sessions needed to improve the required skills					*It is helpful because we sit and we know where we are having the gaps. Okay, so we try to fill those gaps (Midwife, Kenya)*
